# Staff behaviours that promote positive co-worker relationships in health and social care settings: A scoping review with implications for long-term residential care

**DOI:** 10.1016/j.ijnsa.2026.100543

**Published:** 2026-04-19

**Authors:** Kirsty Haunch, Kirstine McDermid, Karen Spilsbury

**Affiliations:** a School of Healthcare, University of Leeds, Leeds, UK; b NICHE-Leeds, University of Leeds, Leeds, UK

**Keywords:** Co-worker relationships, Peer support, Long-term care, Behaviours, Staff, Workforce, Scoping review

## Abstract

**Background:**

Co-worker relationships in long-term residential care play a crucial role in enhancing quality of care. Collaborative relationships among co-workers are consistently linked to positive outcomes in these environments. However, the current evidence on co-worker relationships in long-term residential care is largely descriptive and lacks insights into the behaviours that influence these relationships positively or negatively. Gaining a deeper understanding of these behaviours would support the development of interventions aimed at promoting effective co-worker relationships.

**Aim:**

This scoping review aims to map evidence on staff behaviours that foster positive co-worker relationships and collaboration in health and social care settings, and to interpret these findings for their relevance to long-term residential care.

**Design/Methods:**

We conducted a scoping review to assess the volume, scope and quality of research on co-worker relationships in long-term care, following established guidance. We used the COM-B behaviour change framework, which proposes that behaviour arises from three interacting components: capability (the skills and abilities required to act), opportunity (external factors that enable or hinder action), and motivation (internal processes that direct behaviour). We extracted data from primary studies in broader health and social care settings and mapped findings to COM-B categories. This enabled us to identify behaviours influencing relationships and highlight theories and interventions with potential relevance for long-term residential care. A consultation with long-term care managers was also undertaken to assess the practical relevance of the findings.

**Results:**

Forty-two papers identified behaviours that mapped on to COM-B components: capability (social competence), opportunity (time, space, culture), and motivation (willingness). Consultation with long-term care managers supported the relevance of these findings, highlighting leadership as the most critical factor influencing co-worker relationships. Managers highlighted the importance of leadership behaviours and emphasised the value of strengthening communication and relationship management skills within teams.

**Conclusion:**

By drawing together evidence across health and social care settings, this review advances understanding of how positive co-worker relationships are built and sustained in long-term care residential settings. Our findings position leadership behaviours, not structures or hierarchies, as key mechanisms that shape the relational environment in which care is delivered. Despite this, leadership research in long-term residential remains underdeveloped with few studies specifying actionable behaviours or testing their effects. The practical behaviours we identify offer a foundation for developing relationally focused workforce interventions. Progress depends on coordinated support from employers, commissioners, and policy-makers, along with research investment to evaluate how behavioural approaches can improve staff experiences and improve care quality.

**What is already known**

• Co-worker relationships are central to organisational function and staff well-being in health and social care settings.
• In long-term residential care, co-worker relationships are particularly important due to the demanding nature of the work and limited external support, with peer support often being the primary source of guidance.
• Existing research on co-worker relationships in long-term residential care is largely descriptive and focuses on negative behaviours such as bullying and incivility.

Alt-text: Unlabelled box dummy alt text

**What this paper adds**

• The paper identifies concrete behaviours, such as anticipating others’ needs, emotional regulation, expressing appreciation, and listening actively that promote co-worker relationships, an area overlooked in previous research
• It highlights the pivotal role of leadership behaviours in creating opportunities in which positive co-worker relationships can develop, moving leadership discussions from abstract models to specific, actionable practices
• It offers practical, theory-informed recommendations for workforce development, including communication skills training, relationship focused leadership development, and team structures that support relational practice.

Alt-text: Unlabelled box dummy alt text

## 1 Introduction

Workplace relationships strongly influence job satisfaction, performance, and organisational productivity. They support decision-making, task assistance, innovation, and emotional well-being (Caspar et al., 2020; Güney et al., 2021).

Among these co-worker relationships interactions between peers with no formal authority over one another, are particularly significant as they shape behavioural norms that can promote or hinder engagement and quality of care (Sias, 1995; Sias, 2002). Positive peer relationships enhance morale and meaning at work, while negative experiences such as conflict or incivility remain common (Cooke and Baumbusch, 2021, Güney et al., 2021, Wiener et al., 2009).

## 2 The importance of co-worker relationships in the long-term residential care context

Co-worker relationships are particularly important in long-term residential care. The work in these settings can be rewarding but it can also be difficult as it constitutes physically, mentally and emotionally demanding work (Woodhead et al., 2016). The prevalence of chronic progressive conditions among the population living in long-term residential care means they are older, and living with complex needs and frailty (Dudman et al., 2018). A largely unregistered workforce provides care for this population, with little external support from health and/or social care professionals (Skills for care, 2022).

Care workers, also known internationally as care assistants, certified nursing assistants (CNAs), or nursing aides make up the majority of the long-term residential care workforce (Vellani et al., 2022; Wang et al., 2025). Much of their work, which includes providing personal care, emotional and social support, and supporting meaningful activities, requires them to work long (up to twelve-hour) shifts during a 24-hour period (Ingstad and Haugan, 2024; Vetbuje et al., 2023). This means consistently working unsociable hours and national holidays (Eurofound, 2020; Skills for Care, 2018; Vellani et al., 2022).

Supervision structures vary across long-term residential care settings. In the UK there are two long-term care settings: care homes without nursing (or residential care) and care homes with nursing (or nursing homes). In residential environments, care workers are typically overseen by a senior carer and/or a registered manager, with nursing care and support provided by registered nurses (RN) employed by external primary care services. In nursing homes, an RN acts as the clinical lead, supervises care delivery by care workers, and mentors staff. Medical professionals are not employed by UK long-term residential care settings; instead, medical input comes from visiting primary care practitioners. Consequently, both care workers and RNs rely heavily on peer support within these environments (Enghiad et al., 2022; Skills for Care, 2018).

The long-term residential care workforce is diverse (Bates et al., 2018; Debesay et al., 2022). Staff often differ in educational background, culture and employment status (e.g., overseas workers, agency staff). These differences can enrich team dynamics but also present challenges for communication and collaboration (Roitenberg et al., 2025). Understanding how diversity shapes co-worker relationships is essential for designing inclusive interventions in these environments (Smith et al., 2019).

Despite the demands of the role and level of responsibility, care workers and RNs in long-term residential care settings receive less formal training than equivalent staff in other healthcare settings such as acute and community healthcare services (Meeks et al., 2021). This disparity is evident in countries like the UK, where most long-term residential care, both with and without nursing, is delivered by diverse independent providers, including for-profit, not-for-profit, and charitable organisations (Haunch and Spilsbury, 2025). This fragmented structure leads to variability in training and professional development. Staff often work in isolation, limiting opportunities to build relationships across health and social care (Auschra, 2018; Brennan-Cook, 2020; Liapi et al., 2025). Such isolation fosters organisational silos and reduces integration with wider systems (Barron and West, 2017; Eurofound, 2020). Similar patterns occur in countries with large private long-term residential care sectors, such as the US and Australia, where decentralised governance hinders coordination, restricts shared training, and limits peer support (Colón-Emeric et al., 2014; Edvardsson, 2016; Munkejord and Tingvold, 2019).

While long-term residential care work is undoubtedly challenging, it also offers meaningful and rewarding experiences. Studies have shown that care workers often derive emotional fulfilment from building relationships with residents, contributing to their quality of life, and working within cohesive teams (Madden et al., 2017; Oppel et al., 2019). Understanding how these positive experiences are cultivated is crucial, as they play a significant role in promoting job satisfaction and supporting workforce retention.

The value of relationships in long-term residential care is well recognised. However, research in this field has largely focused on the relationships between staff and residents (Ådland et al., 2022; Haunch et al., 2021; Spilsbury et al., 2024; Surr et al., 2018) and staff and family (Bauer et al., 2014; Haunch et al., 2021; Jones and Moyle, 2016; Tjia et al., 2017). Where co-worker relationships are studied, the work has been descriptive and focused on negative behaviours, such as bullying, incivility and conflict, and the impact for staff and/or residents’ care (Cooke and Baumbusch, 2022; Pickering et al., 2017; Strandmark K et al., 2019). There is limited understanding of behaviours that foster positive co-worker relationships in long-term residential care settings. Some organisational strategies, such as role modelling, flexible working, supervision, and team-building, have been identified (Haunch et al., 2021), but these focus on managerial or organisational levels. Research describing specific behaviours enacted by staff themselves remains scarce, despite the central role of peer support in long-term residential care environments.

## 3 Aim

This scoping review aims to map evidence on staff behaviours that foster positive co-worker relationships and collaboration in health and social care settings, and to interpret these findings for their relevance to long-term residential care.

## 4 Methods

### 4.1 Study design

A scoping review to identify, examine and synthesise published evidence associated with co-worker relationships in health and social care, and to establish implications of the findings for co-worker relationships in long-term residential care. This was considered the most appropriate review method to determine the volume and scope of research in this field. Although the review drew on studies from various health and social care settings, synthesis focused on behaviours relevant to long-term residential care due to the limited number of long-term residential care specific studies and the potential applicability of findings from other contexts. It therefore identifies behaviours that promote co-worker relationships across health and social care, highlighting transferable insights for long-term residential care environments.

We followed the six-stage approach described by Arksey and O’Malley (Arksey and O'Malley, 2005), and Levac et al. (Levac et al., 2010). In the first stage, we defined the research question: what staff behaviours promote positive co-worker relationships across health and social care and what is their relevance for long-term residential care?; Stages 2 to 6 [(2) searching for relevant studies; (3) selecting studies; (4) charting the data; (5) collating, summarising and reporting the findings; and (6) consulting with stakeholders] are documented in detail below.

The PRISMA extension for scoping review guidance (PRISMA-ScR) was used for reporting (Tricco et al., 2018). The protocol was registered on the Open Science Framework repository (Haunch, 2021).

## 5 Search strategy (stage 2)

The search strategy was developed in consultation with an information specialist. Relevant studies were identified by defining key concepts, locating and using relevant sources, using appropriate search terms, time frame and languages (Table 1).

**Table 1:** Overview of search strategy.

Searching for studies	Description
Key concepts	Co-worker relationships The term 'co-worker relationships' refers to individuals who work in a similar role (for example RN or care worker) or have a similar level of responsibility at unit level (including unit level supervisors).
Sources of information	Published or unpublished primary studies, theses/dissertations, theoretical discussions and grey literature. Grey literature was sourced from the following websites: Agency for Healthcare Quality and Research (AHQR); American Nurses Association (ANA); Academy of Management; My Home Life; Care Quality Commission (CQC); Skills for Care.
Databases	A range of electronic databases (including Ovid MEDLINE, PsycINFO, EMBASE, CINAHL, Cochrane Databases of Systematic Reviews, Web of Science and Scopus) were used to search for literature, as well as citation searches. Several websites were also targeted for unpublished literature, such as the Agency for Healthcare Research and Quality (AHRQ), American Nurses Association. Academy of Management and other sites were identified by the research team and information specialist.
Time span	All literature database searches were searched from 1990 to June 2024. An updated search was performed in October 2025 (Supplementary Table 5).
Search terms	An information specialist (KM) supported the design of the search strategy. There were three concepts: (1) co-worker relationships; (2) quality of care (QOC), quality of life (QOL) and quality of work (QOW) work; and (3) health and/or social care setting. The full search terms, including Boolean operators, database-specific syntax, and search strings for all databases, are provided in Supplementary Materials 1**.**

We did not apply a PCC framework limited to long-term residential care, as this would have excluded relevant literature. Instead, we used broader inclusion criteria to capture intra-professional co-worker relationships across health and social care. This aligns with scoping review methodology, which supports mapping concepts across diverse contexts. Relevance to long-term residential care was assessed through theory-informed synthesis and stakeholder consultation, identifying behaviours with practical applicability to residential care.

Full search strategies for all databases, including exact search strings and Boolean logic, are provided in Supplementary Materials 1 and 5**.** Grey literature sources consulted are also listed and described.

## 6 Study selection (stage 3)

**Fig. 1:**
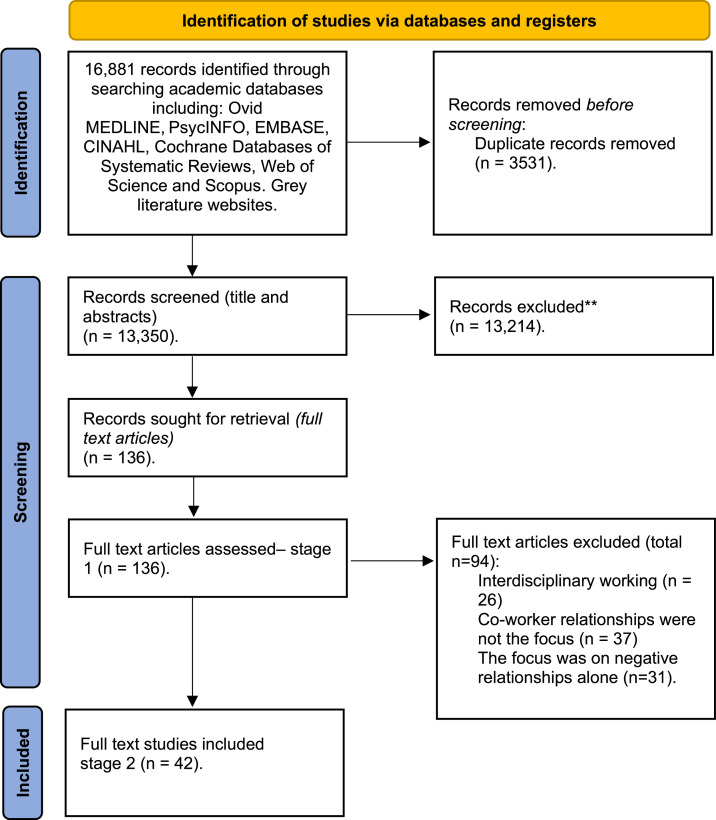
PRISMA flow diagram of study selection, adapted from Tricco et al. (2018).

A total of 16,881 records were identified through database searching. After removing 3531 duplicates, 13,350 abstracts remained for title and abstract screening. Of these, 13,214 records were excluded based on the inclusion/exclusion criteria. This process resulted in 136 full-text articles being assessed for eligibility (Fig. 1). Studies were included for full text review if they focused on:

1. Intra-professional co-worker relationships;
2. Relationship building behaviours;
3. Quality of life, care and work;
4. Health and/or social care setting;
5. Published or unpublished primary study, a thesis/dissertation, a theoretical discussion or grey literature.

Studies were excluded if they focused on:

1. Inter-professional, multidisciplinary co-worker relationships, as these relationships were beyond the scope of this review;
2. Co-worker relationships were not the main focus of the paper, as such studies were considered too far removed from our area of interest;
3. The focus of the paper was solely negative co-worker behaviours (such as bullying, incivility, conflict or abuse). This was because the purpose of the review was to identify behaviours that actively promote positive co-worker relationships in long-term residential care. Studies examining negative behaviours alone do not provide data on constructive or relationship-building mechanisms, and their inclusion would have shifted the focus of the synthesis away from the review aim. Given that negative behaviours have been extensively examined in previous literature, excluding these studies enabled the review to focus on the comparatively under-explored evidence concerning positive staff behaviours relevant to co-worker relationships.

Following full-text review, 94 articles were excluded for the following reasons: inter-professional focus (n = 26), co-worker relationships were not the main focus (n = 37), or the focus was only on negative behaviours (n = 31). There were 42 studies included in the final synthesis (Fig. 1). The PRISMA flow diagram (Fig. 1) was adapted from Tricco et al. (2018) and follows the PRISMA-ScR guidelines.

Study selection occurred in two stages (Fig. 1). The lead reviewer (KH) screened all titles and abstracts, with a second reviewer (KS) checking 10% for consistency; discrepancies were resolved through discussion. Both reviewers then screened full texts, and two papers requiring further discussion were included in the synthesis.

### 6.1 Included studies

Forty-two studies were included. Eighteen were quantitative studies: randomised controlled trials (n = 1), quasi experimental design (n = 1), pre-post study designs (n = 6), and cross-sectional surveys (n = 10). Fourteen had a qualitative design: interviews/focus groups or observation (n = 14). One was a mixed methods study. There were four literature reviews, two theoretical discussion papers of co-worker relationships and three opinion papers.

Most of the included studies were conducted in a hospital setting (n = 31), followed by long-term residential care (n = 7), and primary care (n = 1). Twenty-nine studies focused on RNs or licensed practical nurses (LPNs). Other studies focused on medical practitioners/doctors (n = 2), care workers (n = 1), or both RNs and care workers (n = 7). The three opinion papers focused on RNs in hospital settings.

Date of publication ranged from 1990–2023. Most studies were from North America (n = 21), with the remaining papers from Europe (n = 11), Australia/New Zealand (n = 3), Asia (n = 3) and Turkey (n = 1). The three opinion papers were from North America.

The included papers focused on the following themes related to co-worker relationships: teamwork, communication, social capital, group cohesion, social support, co-worker trust, resilience, culture, reciprocity, collegiality, and relations.

## 7 Charting data (stage 4)

The characteristics of included papers were charted. Extracted data included: year, authors, publication title, research question or study purpose, study design, context (including country), participants, sample size, theoretical/conceptual framework, intervention (type, elements), definitions of concepts, data collection methods, relevant findings, and discussion. The lead author extracted data from all included studies, with a second author (KS) double checking 10% of the extraction for consistency.

## 8 Collating, summarising and reporting findings (stage 5)

Evidence was first collated and summarised according to: overall number of studies, concepts studied, types of study design, setting and participants, date published and country of origin*,* tools that measure co-worker relationships and interventions used to promote co-worker relationships. We used a behaviour change theory, capability, opportunity, motivation, behaviour (COM-B) (Michie et al., 2011), as a deductive theoretical framework to guide the synthesis of behaviours identified across the included studies. COM-B posits behaviour as an interaction between capability, opportunity, and motivation (Michie et al., 2011). A particular behaviour occurs when the person has the capability and opportunity to act and is more motivated to perform that behaviour than any alternative. Over time, increased capability and opportunity strengthen motivation and the likelihood of the behaviour. Behaviour then feeds back to all three components creating positive effects, in this case collaborative co-worker relationships, or negative effects, conflict, bullying (Michie et al., 2011).

The COM-B model has been effectively applied in health and social care research, but not previously applied in scoping reviews synthesising evidence on co-worker relationships. Rather than a coding tool, we used COM-B to organise and interpret behaviours by underlying mechanisms. Behaviours were mapped to capability, opportunity, or motivation, reflecting skills and competencies, environmental and organisational factors, or internal drivers such as values and willingness. This theory-informed approach structured the synthesis and enhanced its explanatory power.

Behavioural synthesis was conducted by one reviewer (KH), who mapped the extracted behaviours from included studies onto the COM-B framework, in consultation with a second reviewer (KS).

To guide the synthesis of behaviours identified across the included studies, there were three structured steps:

a. defining the focus for change as “relationship-building behaviours” relevant to co-worker interactions;
b. identifying and selecting target behaviours from the included studies; and mapping these behaviours onto the COM-B domains based on their underlying mechanisms (Braun and Clarke, 2006; Clarke and Braun, 2013);
c. Describing target behaviour(s) in detail and in their surrounding context.

In stage 6 we consulted long-term residential care managers to understand which target behaviours were most important to achieve change from their perspective.

## 9 Consultation with stakeholders (stage 6)

The stakeholder consultation was undertaken to explore the prioritisation and practical relevance of the synthesised behaviours, in line with scoping review guidance, and did not involve the collection of primary research data.

Behavioural analysis requires the prioritisation of behaviour(s) to target. Starting with a small number of behaviours is recommended in the COM-B model to facilitate incremental change, building on success, rather than trying to enact rapid changes (Michie et al., 2011). Prioritising behaviours and identifying what needs to change was undertaken in consultation with stakeholders (Buus et al., 2022; Michie et al., 2011), following the review findings.

Stakeholder consultation was conducted in line with scoping review guidance to enhance the relevance and applicability of findings. Long-term residential care managers (n = 6) were a mixture of RNs (n = 1) and non-clinical managers (n = 5) from a medium sized, for-profit long-term residential care organisation. The stakeholder consultation was limited to a small group of six managers from a single organisation due to resource limitations. We consulted with managers in long-term residential care about the review findings, using a series of prompts (Table 2).

**Table 2:** Consultation with long-term residential care managers question prompts.

Question prompts for long-term residential care managers Do the findings resonate with you? Is there anything you disagree with? Is there anything missing? In your experience, which is the most important contributor to good co-worker relationships in your home? Where would you start if you wanted to make a change to co-worker relationships in your team? Which behaviour do you perceive is the easiest to change and would have the most impact? How would you measure change in co-worker relationships?

## 10 Quality assessment of studies

Although quality appraisal is not required in scoping reviews, we undertook methodological appraisal to support cautious interpretation of the evidence and to provide transparency about the strengths and limitations of the included studies. Studies (n = 39) were quality assessed using the Mixed Methods Appraisal Tool (MMAT) and AMSTAR 2 tool (Hong et al., 2018; Shea et al., 2017). One reviewer (KH) independently screened and assessed methodological quality with a second reviewer (KS) checking 50% of the papers (Supplementary Materials 2). Explicit notes were recorded where criteria could not be judged with confidence, rather than assigning affirmative ratings. Any disagreements were resolved through discussion. Opinion papers (n = 3) were given less weight in the narrative and were cited to strengthen an idea made by primary research papers, rather than adding standalone ideas or data.

Quality appraisal was conducted to support interpretation of the findings rather than to exclude studies. Of the 42 studies included in the review, 35 empirical studies were appraised using the Mixed Methods Appraisal Tool (MMAT) (Hong et al., 2018) and four were appraised using AMSTAR (Shea et al., 2017). Three opinion papers were not appraised, as the MMAT is not designed for non-empirical study types.

## 11 Ethical considerations

Ethical approval was not required as this was a consultation activity, not primary research (Arksey and O'Malley, 2005). Participants were informed of the purpose, contributions were voluntary, and no personal or sensitive data were collected. Discussions were not recorded. The group provided perspectives on the relevance of findings for long-term residential care, including which were most important. Consultation findings are reported after the review results.

### 11.1 Behavioural analysis of review findings

a. Relationship building: the focus for change

**Fig. 2:**
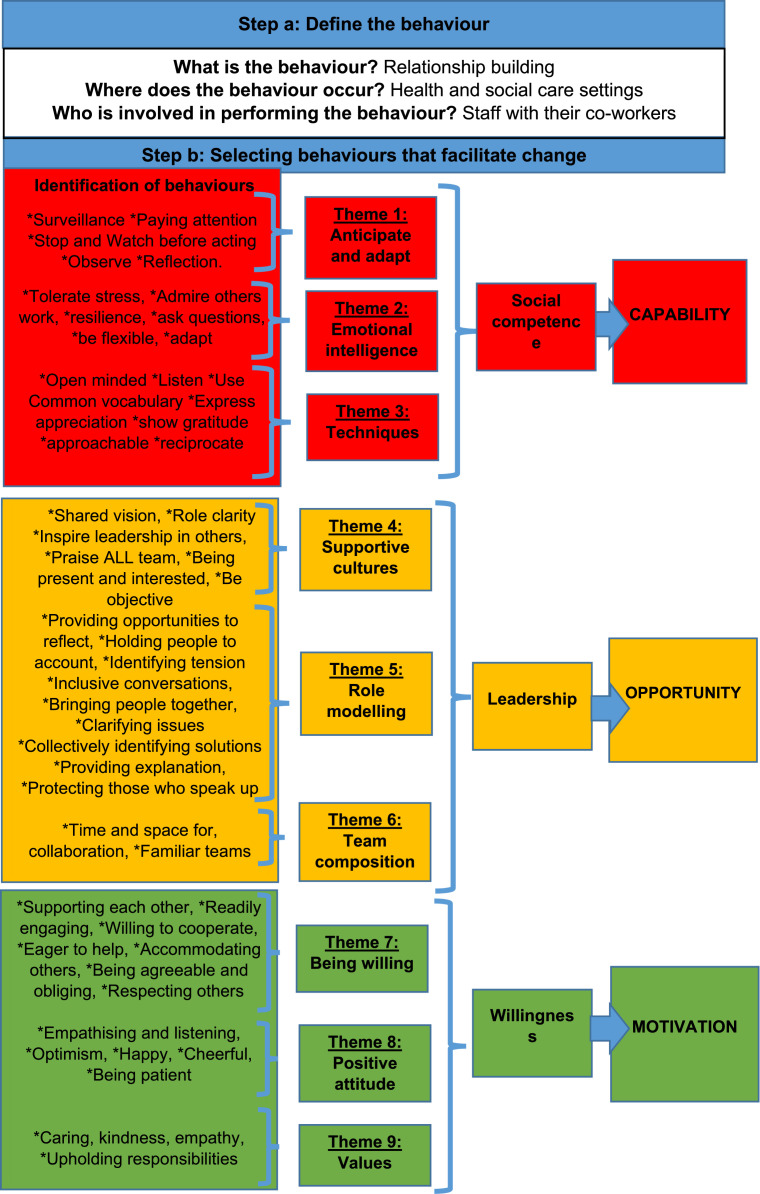
Behavioural analysis of staff behaviours promoting co-worker relationships, organised using the COM-B framework.

Relationship building was identified as the focus for change. Our analysis concentrated on identifying behaviours reported in the included studies that promote positive co-worker relationships (Fig. 2).

b. Identifying and selecting target behaviours and mapping to COM-B

We identified 51 relationship building behaviours reported the included empirical studies and mapped these to COM-B domains. Table 3 illustrates how behaviours were extracted from the literature, grouped into sub-themes, and assigned to the capability, opportunity, or motivation categories, with illustrative quotes drawn from the original papers, Supplementary Table 4 shows which studies contributed to each COM-B domain. This mapping reinforces the transparency and rigour of our synthesis process.

**Table 3:** Examples illustrating how specific behaviours were classified under the COM-B domains.

Quote	Behaviour identified	Sub theme	Theme	Mapped on to COM-B
As reported by Duddle et al. (2007): ‘‘Some participants in our study had clearly been able to move on from the negative emotional experiences in their workplace. They had been able to adapt and persevere in the face of conflict, both around them and directed at them. This ability to be resilient or to bounce back helps individuals cope with adverse or changing circumstances” (Duddle and Broughton, 2007), page 34).	Resilience	Emotional Intelligence	Social competence	Capability
As reported by Kim and Oh (2016): “Nurses became familiar with hospital work and were simultaneously asked to quickly adapt into the culture of their new ward, was the time during which nurses began to learn the unspoken rules of their new environment. In addition to the ways in which specific clinical duties should be performed, unspoken rules of behaviour between nurses constituted an important part of ward culture. From this perspective, the major goal for newly hired nurses to achieve in this phase was to learn the unspoken rules as well as what was unacceptable; new nurses learned about unacceptable communication and behavioural patterns as a member of the specific unit” (Kim and Oh, 2016, page 6).	Paying attention	Anticipate and adapt	Social competence	Capability
As reported by Colón-Emeric et al. (2014): “Be open, listen, and respond to what people say, hear with thoughtful attention, and make a conscious effort to stop, watch, and act” (Colón-Emeric et al., 2014, box 1, page 449).	Stop and watch	Anticipate and adapt	Social competence	Capability
As reported by Padgett (2013): “Without a language for professional group practice (denoting shared decision-making and responsibility), the staff on the unit identified themselves simply as people who helped each other and got along well” (Padgett, 2013, page 9).	Shared vision	Supportive culture	Leadership	Opportunity
As reported by Thomas and Galla (2013): “The team member being addressed must acknowledge [the concern]. If the outcome is still not acceptable take a stronger course of action and/or utilise a supervisor or chain of command. Empowers all team members to ‘stop the line’ if they sense or discover an essential safety breach” (Thomas and Galla, 2013; Table 1, page 429).	Holding people to account	Role modelling	Leadership	Opportunity
As reported by Campbell et al. (2020): “Role clarity is an important component of effective delegation. If RNs and NAs struggle in silos, they will be unable to bridge the gaps in patient care. Nurse managers must facilitate effective delegation practices between RNs and NAs while maintaining relational quality between the RN and NA” (Campbell et al., 2020, page 1470).	Role clarity	Supportive cultures	Leadership	Opportunity
As reported by Padgett (2007): “The more positive way would be to say: I will respect the judgments of my fellow nurses, and I expect them to give me that respect in return.” (Padgett et al., 2007, page 1412).	Respect	Being willing	Willingness	Motivation
As reported by Jakobsen et al. (2018): “Having a positive attitude towards work and a good companionship with colleagues may make these hassles bearable and work more enjoyable” (Jakobsen et al., 2018, page 1132).	Positive mood	Positive attitude	Willingness	Motivation
As reported by Schirm et al. (2000): “If you don’t have the caring and willingness to show respect, you shouldn’t even get into the field because it’s something that has to be within you” (Schirm et al., 2000, page 284).	Caring, respect	Values	Willingness	Motivation

The stakeholder consultation was used to explore the prioritisation and practical relevance of these behaviours within long-term residential care and did not contribute additional behavioural data to the synthesis.

Table 3 presents illustrative verbatim quotations drawn directly from the included empirical studies. These quotations are used to demonstrate how behaviours were extracted from the literature, grouped into sub-themes, and mapped to the capability, opportunity, or motivation components of the COM-B framework. All quotes originate from the primary studies included in the review and do not derive from the stakeholder consultation.

Fig. 2 provides an illustrative overview of each behaviour mapped onto the COM-B domains based on their underlying mechanisms.

c. Describing target behaviour(s) in detail and in their surrounding context

The following sections describe each target behaviour in detail and its surrounding context, organised according to the COM-B domains: capability, opportunity, and motivation.

## 12 Social competence (capability)

Social competence is a ‘skill’ characterised by an ability to anticipate and adapt to situations, show emotional intelligence and use techniques to promote relationship building. Social competence was mapped on to the capability component of the COM-B framework.

### 12.1 Anticipate and adapt

Social competence is the ability to anticipate and adapt to social situations, recognise others’ feelings and intentions and select appropriate behaviours’ (Hoare et al., 2013; Kalisch et al., 2010; Yan et al., 2022). In dynamic health and social care settings, personality clashes and conflict are common (Norikoshi et al., 2018). Socially competent employees are resilient and able to manage difficult interactions effectively (Duddle and Boughton, 2007; Kaya and Eskin Bacaksiz, 2022; Kim and Oh, 2016). Developing communication skills to handle challenging situations is a key goal of interventions aimed at promoting care quality and staff well-being (Clark et al., 2022; DiMeglio et al., 2005; Kaya and Eskin Bacaksiz, 2022; Kile et al., 2019; Lux et al., 2014).

### 12.2 Emotional intelligence

Social competence is closely linked to emotional intelligence (Duddle and Boughton, 2007; Lux et al., 2014; Quoidbach and Hansenne, 2009). Emotionally intelligent staff tolerate stress and frustration, recognise their limits, and seek help when needed (Duddle and Boughton, 2007; Quoidbach and Hansenne, 2009). Regulating emotional reactions helps resolve difficult situations without outbursts, preserving co-worker relationships (Duddle and Boughton, 2007; Kaya and Eskin Bacaksiz, 2022; Yan et al., 2022). Teams with higher emotional intelligence manage conflict constructively, while those with lower levels tend to avoid it (Lux et al., 2014). Reviewing feedback as an opportunity rather than criticism is another key behaviour (Quoidbach and Hansenne, 2009).

### 12.3 Techniques

Social competence involves observing context and responding thoughtfully in challenging interactions. Rather than criticising, socially competent staff seek to understand colleagues (Colón-Emeric et al., 2014; Moore et al., 2019). Key behaviours include: open-mindedness, active listening, adaptability, and providing support; their absence often leads to personality clashes (DiMeglio et al., 2005; Emich, 2018; Schirm et al., 2000; Velando-Soriano et al., 2020; Winning et al., 2018; Yan et al., 2022). Expressing appreciation, thanking, praising, and acknowledging contributions, strengthens cooperation and team cohesion (Colon-Emeric., 2014; Norikoshi et al., 2018; Padgett, 2013; Potter and Grant, 2004).

Social competence develops through practice and experiential learning, supported by experienced staff and caring cultures. Education alone is insufficient (Bellury et al., 2016; Duddle and Boughton, 2007; Kim and Oh, 2016).

The behaviours associated with social competence, such as anticipation, adaptability, emotional intelligence, and relationship-building techniques, are grouped under the ‘capability’ domain in Fig. 2, and further organised into three sub-themes: (1) anticipate and adapt, (2) emotional intelligence, and (3) techniques.

## 13 Leadership (opportunity)

Leadership is made up of: supportive cultures, role modelling and team composition and is conceptualised as an ‘opportunity’ component in the COM-B framework. Whilst individuals influence culture, organisational leaders set the tone for interactions within teams as explained in this section.

### 13.1 Supportive cultures

Culture reflects the social environment where staff interact and share ideas (Heponiemi et al., 2012). Studies describe ‘unspoken rules’ shaping norms of support (Duddle and Boughton, 2007; Kim and Oh, 2016; Madden et al., 2017). In caring cultures, these norms include enthusiasm, praise, listening, and information sharing (Barry et al., 2019; Clark et al., 2022; Toles and Anderson, 2011). Staff in such environments report feeling (Madden et al., 2017) safe, heard, trusted and able to learn from one another, describing teamwork as ‘smooth’ or ‘like dancing (Campbell et al., 2020; Duddle and Boughton, 2007; Lehmann-Willenbrock et al., 2012).

Supportive team cultures help staff to navigate day-to-day challenges and deliver care effectively (Duddle and Boughton, 2007). Such environments foster positive attitudes, ease stress for new staff, prevent conflict, and make work more enjoyable (Clark et al., 2022; Jakobsen et al., 2020). They also help manage negative experiences (Lux et al., 2014). Staff often describe this as ‘moral support’, viewing co-workers as their most important resource (Madden et al., 2017; Norikoshi et al., 2018).

Unsupportive environments foster behaviours that undermine relationships, including criticism, ignoring, defensiveness, intimidation, and a lack of support, for new staff members (Clark et al., 2022; Padgett, 2013; Pedersen et al., 2023). Such settings are often described as unwelcoming, with rudeness and poor supervision (Bellury et al., 2016; Colón-Emeric et al., 2014). These conditions create a ‘sink or swim’ culture, reducing staff motivation and an ability to build positive relationships (Duddle and Boughton, 2007).

### 13.2 Role modelling

Leaders set the tone and establish group norms (Clark et al., 2022; Kile et al., 2019; Pedersen et al., 2023) by role modelling expected behaviours, enthusiasm, praise, listening, and inclusive communication (Campbell et al., 2020; DiMeglio et al., 2005; Madden et al., 2017). They minimise conflict by identifying tension, facilitating dialogue, and monitoring progress (Barry et al., 2019; DiMeglio et al., 2005; Kile et al., 2019; Thomas and Galla, 2013). Effective leaders reward staff who speak up, address bullying, and hold individuals accountable for collaborative behaviours (Hofmeyer and Marck, 2008). These actions create shared expectations that enable teams to function cohesively (Bellury et al., 2016; Kalisch et al., 2010).

Leaders who model collaborative behaviours are relationship oriented, focusing on team satisfaction, motivation, and well-being (Clark et al., 2022; Pedersen et al., 2023). They are present, supportive and recognise individual expertise, framing feedback constructively (Norikoshi et al., 2018; Padgett, 2013). Leaders also inspire others by encouraging experienced staff to mentor newcomers, creating a positive feedback loop of role modelling behaviours (Clark et al., 2022; Moore et al., 2019). Strategies include explaining issues during tension, helping staff view situations objectively, and providing opportunities for reflection (DiMeglio et al., 2005; Kaya and Eskin Bacaksiz, 2022; Madden et al., 2017).

Promoting role clarity among team members is important. Collaboration is stronger when staff not only understand their own roles but also each other’s (Campbell et al., 2020; Kalisch et al., 2010; Moore et al., 2019; Schirm et al., 2000). Knowing the roles and skills of each member of the team, for example, minimises confusion, misunderstandings and assumptions (Kalisch et al., 2007). It also allows for contacting the person best equipped to aid in solving particular problems (Barry et al., 2019; Emich, 2018; Schirm et al., 2000). Role ambiguity and unsupportive leadership creates confusion and conflict (Barry et al., 2019; Havig et al., 2013; Moore et al., 2019).

Establishing role clarity and embedding a shared sense of work expectation has been associated with better uptake of evidence based practice guidelines (Toles and Anderson, 2011), improved quality of care (Campbell et al., 2020; Colón-Emeric et al., 2014; Havig et al., 2013; Toles and Anderson, 2011), and staff intention to stay (Brunetto et al., 2013; Toles and Anderson, 2011). In the absence of shared expectations and understanding, staff identify themselves simply as people who help each other out and get along well ‘at times’ (Havig et al., 2013; Padgett, 2013).

As well as setting expectations through relational support, leaders are also described to create the physical opportunities that promote collaborative relationships, the composition of teams is key.

### 13.3 Composition of teams

Team composition provides important foundations for building collaborative and positive co-worker relationships.

Developing small, consistent teams is an effective strategy to enhance teamwork and communication (Campbell et al., 2020; Havig et al., 2013; Potter and Grant, 2004). Employees in these teams gain a deep understanding of each other's strengths, weaknesses, and unique traits, fostering close relationships that provide valuable support (Campbell et al., 2020; Havig et al., 2013; Munkejord, 2019; Potter and Grant, 2004). Implementing 'buddy systems', where staff members are paired to discuss work activities, can further strengthen co-worker relationships, build trust, and boost confidence among team members (Munkejord et al., 2019).

As team size grows, the likelihood of collaborative behaviours decreases and it is harder for staff to get to know each other, particularly with staff turnover (Havig et al., 2013; Kalisch and Begeny, 2005; Potter and Grant, 2004). It is therefore important that there is an opportunity for staff to get to know each other.

Sharing observations during the day helps prevent isolation and unresolved conflict (Roth and Markova, 2012). Regular opportunities for communication, through meetings, handovers, huddles, or debriefs, enable staff to clarify messages, optimise schedules, and negotiate workloads, improving care quality and safety (Clark et al., 2022; Jakobsen et al., 2020; Killmeck., 2022). Formal and informal strategies include inclusive handovers, extended meetings with social time, structured walk rounds, and open forums for reflection (Bellury et al., 2016; Colón-Emeric et al., 2014).

## 14 Willingness (motivation)

Willingness is internal to each individual person and therefore conceptualised as a ‘motivation’ component in the COM-B framework. Willingness is made up of being willing, positive attitude and caring values.

### 14.1 Being willing

Central to collaborative co-worker relationships are people [staff] who are ***willing*** to emotionally and physically support one another when providing care (Campbell et al., 2020; Emich, 2018; Madden et al., 2017; Potter and Grant, 2004). Being willing means readily engaging in extensive informal communication (Padgett, 2013), teamwork (Colón-Emeric et al., 2014), cooperation (Madden et al., 2017), and civility (Clark et al., 2022). The behaviours of a 'willing' staff member, as shown in Fig. 2 (Emich, 2018; Norikoshi et al., 2018; Schirm et al., 2000), lead to better care for hospital patients (Colón-Emeric et al., 2014) and are seen as improving the quality of care for residents in long-term care settings (Madden et al., 2017; Schirm et al., 2000; Toles et al., 2011).

### 14.2 Positive attitude and caring values

‘Being willing’ relates to a positive attitude (Duddle and Boughton, 2007; Emich, 2018; Quoidbach and Hansenne, 2009) and a set of underlying caring values such as kindness, compassion and respect (Bellury et al., 2016; Norikoshi et al., 2018). Collectively, being willing, positive attitude and caring values are associated with having a positive effect on collaboration (Colon-Emeric et al., 2014; Ericson-Lidman et al., 2015).

Staff working in health and social care settings often share their emotions during daily tasks; this can significantly influence the ‘mood’ of staff both positively and negatively (Colon-Emeric et al., 2014). Motivation and willingness can create a positive working atmosphere, making workplace challenges easier to navigate and work more enjoyable (Duddle and Boughton, 2007; Emich, 2018; Potter and Grant, 2004; Schirm et al., 2000).

When staff do not emotionally or physically support one another, this may reflect a lack of willingness or an inability to do so, shaped by communication challenges, uncertainty about roles or expectations and the diverse educational and cultural backgrounds of the workforce. As a result, co-worker relationships are described to be non-existent, leading cultures of silo working (Madden et al., 2017). A number of studies describe silo working (Colón-Emeric et al., 2014; Duddle and Boughton, 2007; Jakobsen et al., 2018; Madden et al., 2017), characterised by: working alone, excluding co-workers from decisions, deviating from care plans, not asking for advice and shirking responsibilities (Campbell et al., 2020; Madden et al., 2017). This results in negative attitudes, a lack of trust and not feeling able to speak up (Colón-Emeric et al., 2014; Duddle and Boughton, 2007; Jakobsen et al., 2018; Madden et al., 2017). Some staff also express resentment towards unmotivated and unwilling co-workers (Emich, 2018; Schirm et al., 2000).

## 15 Summary of review findings

In summary, our review suggests co-worker relationships are more likely to develop and be sustained if

Team members are socially competent (i.e., they are capable);

- Staff teams are given the time and space to collaborate in caring cultures (i.e., are given the opportunity by leaders in their organisation);
- Staff want to form co-worker relationships. That is they are willing, have a positive attitude and have caring values (motivated).

## 16 Consultation findings (stage 6)

In this section we focus on the relevance of the review findings for long-term residential care settings, prioritising, where possible, which behaviours are most important in long-term residential care.

The themes of social competence, leadership and willingness all resonated with long-term residential care managers. Leadership behaviours were perceived as having the greatest impact on positive co-worker relationships. Managers reiterated that care workers learn on the job, with minimal formal communication training. Skills, confidence and motivation develop over time, with support from experienced staff. Managers rely on competent team members to mentor new workers and want leadership development within teams as a priority.

Box 1 illustrates that long-term residential care managers suggest leadership is a priority to promoting positive co-worker relationships.

**Box 1:** Long-term residential care manager perspectives on COM-B themes.

Leadership behaviour	Description
Encouraging leadership at all levels	Actively empowering staff at all seniority levels to take initiative, contribute ideas, and role model positive behaviours; creating opportunities for peer learning and shared responsibility for maintaining a constructive workplace culture.
Creating supportive leadership mechanisms	Ensuring new staff are supported through structured on-boarding, buddying or mentoring with experienced colleagues, protected time for learning, and regular check-ins to prevent early overwhelm or failure.
Setting the tone	Demonstrating and consistently reinforcing best practice through visible role modelling, clear expectations, and ongoing communication about professional standards and desired team behaviours.
Spotting talent	Identifying staff strengths early and providing developmental opportunities such as additional responsibilities, training, coaching, or progression pathways to help individuals grow and contribute more fully.

## 17 Discussion

This scoping review identified and synthesised 51 behaviours that promote positive co-worker relationships in health and social care settings, interpreted through the COM-B behaviour change model. Across the 42 included studies, three mechanisms consistently supported co-worker relationships: social competence (capability), leadership-created opportunities (opportunity), and willingness (motivation). Consultation with long-term residential care managers confirmed these themes and highlighted leadership behaviours as the most influential for everyday relational practice.

Previous research in long-term residential care has focused predominantly on negative workplace behaviours such as bullying, incivility, and conflict, our review shifts the emphasis toward identifying constructive, malleable behaviours that can be supported, practiced and reinforced. This provides a new perspective for a sector in which peer support is important, formal training is limited, and relationships between co-workers strongly shape care quality and staff wellbeing.

How our findings extend existing knowledge

1. Social competence as a core behavioural capability for collaborative co-worker relationships

Across the included studies, social competence emerged as fundamental to collaborative co-worker relationships. Behaviours such as anticipating others’ needs, adapting communication, regulating emotions, asking questions, expressing appreciation and pausing before responding, consistently supported collaboration. Although elements of social competence are discussed in broader healthcare literature (Benington et al., 2020; Cheshire et al., 2020; Turnpenny, 2021), they have not previously been synthesised as a coherent behavioural capability specifically underpinning co-worker relationships in long-term care.

This review shows that social competence is not a peripheral ‘soft skill’, but a central element of effective teamwork in relationally intense environments. Identifying social competence as a core capability highlights a practical area for development, particularly given many staff learn communication and teamwork skills informally. Embedding structural interpersonal skills training into induction, supervision, and ongoing development could strengthen co-worker relationships, and enhance high quality care.

2. Leadership behaviours create the opportunities for collaboration

Leadership behaviours were consistently identified from the included studies as creating the opportunities and conditions in which positive co-worker relationships can develop. These included: role modelling inclusive communication, setting clear expectations, facilitating problem-solving conversations, enabling reflection and establishing small consistent teams. These behaviours shaped the relational environment and influenced whether constructive collaboration could take place.

Stakeholder consultation reinforced these findings. Managers emphasised that visible leadership, early support for new staff, and consistent modelling of respectful communication had the most influence on team functioning. Focusing on these specific, observable leadership behaviours, rather than abstract theoretical models (Kuluski et al., 2021; Wang et al., 2022; Zonneveld et al., 2021), provides a practical route to improving teamwork, supporting workforce stability, and strengthening care quality.

3. Willingness as a motivational driver of everyday relationship building

Willingness, expressed as kindness, respect, positive attitudes, eagerness to help, and readiness to engage, was a key motivational mechanism underpinning positive co-worker relationships. When willingness was present staff were more cooperative, communicative, and supportive; when absent, silo working and interpersonal avoidance were described.

Our synthesis shows that willingness is shaped by capability and opportunity, rather than being a fixed individual trait. Supportive leadership, clear roles, consistent team, and relational competence all influenced whether staff felt motivated to engage positively with colleagues. Recognising willingness as modifiable highlights an achievable organisational strategy for strengthening team cohesion in long-term residential care.

What this review contributes

This review makes three key contributions:

• It identifies specific, positive, relationship building behaviours, addressing a gap in a literature that has largely focused on negative co-worker interactions;
• It offers a theory-driven behavioural explanation (via COM-B) for how co-worker relationships form and how they can be strengthened in practice;
• It demonstrates that leadership behaviours are the primary mechanism through which capability (social competence) and motivation (willingness) are be enacted in daily work.

Together, these contributions move beyond descriptive accounts of workplace culture by providing an actionable behavioural perspective.

### 17.1 Implications for practice

The findings suggest several practical implications for long-term residential care:

• Leadership development should prioritise relational behaviours, including setting expectations, providing support, modelling respectful interactions, and addressing tensions constructively;
• Team structures should support collaborations, such as small consistent teams, buddying arrangements, and protected time for shared reflection;
• Social competence training, including communication, emotional regulation, situational awareness, and appreciation techniques, should be incorporated into induction and ongoing development;
• Workplace cultures should reinforce willingness by recognising supportive behaviours and attending to staff emotional needs.

These recommendations provide realistic, practical strategies aligned with workforce challenges in long-term residential care.

### 17.2 Implications for policy

Policy frameworks should recognise that relational capability is integral to care quality. Support for leadership development, communication and relationship management training and reflective practice structure may improve teamwork and staff stability.

Policy frameworks should acknowledge workforce diversity and support managers to build inclusive, psychologically safe environments in which positive co-worker relationships can develop.

### 17.3 Implications for research

Future research should develop and evaluate behaviourally specified interventions that target:

• Specific leadership practices;
• Components of social competence;
• Motivational strategies that support willingness.

Interventions should be grounded in COM-B, and adapted for the realities of long-term residential care workforces.

### 17.4 Strengths and limitations

This review provides a theory-informed synthesis of behaviours that promote positive co-worker relationships. Limitations include variation in definitions of co-worker relationships and the predominance of hospital-based publication bias. These factors should be considered when interpreting generalisability.

We provided detailed information on included studies to enable assessment of relevance and limitations. The initial searches were completed up to June 2024, with an update in October 2025 identifying two new papers (Varga et al., 2025; Zettna et al., 2025). Both reinforced our findings, particularly the importance of interpersonal dynamics and leadership behaviours, so were not included in the synthesis (Stokes, Sutcliffe and Thomas, 2023). To support transparency and replication, the full search strategy is available in Supplementary File 5.

The terms co-worker and relationships are vast and poorly defined in the literature. We derived our search terms from identified literature, so there is a possibility that we may have missed key papers. We may have also missed some relevant information from regulatory reports, sector led work, practice journals and/or other grey literature. Despite the breadth of our search, the final selection may be subject to bias, including publication bias, overrepresentation of hospital-based studies, and underreporting of negative findings. These factors should be considered when interpreting the generalisability of our findings

Screening and data extraction were primarily conducted by one reviewer, with limited checks by a second reviewer. While this ensured consistency, it introduces a potential risk of bias that should be considered when interpreting the findings. The consultation involved six managers from one long-term residential care provider, excluding frontline staff. This limited sample may not represent sector-wide views. Its purpose was to check the relevance of review findings and provide context, not to generate new data or influence theme prioritisation.

Overall, the methodological quality of included studies was good, with most studies clearly describing their aims, data collection methods, and analyses. However, the evidence base was predominantly descriptive and cross-sectional, with few intervention studies and limited use of longitudinal or experimental designs. As a result, findings primarily reflect associations and reported experiences rather than causal effects. Quality appraisal informed the interpretation of findings rather than study inclusion or exclusion. Studies with stronger methodological designs were prioritised when drawing conclusions about consistent behavioural patterns, while findings from opinion papers and methodologically weaker studies were used to support or illustrate themes rather than generate them.

## 18 Conclusion

This review provides a theory-informed synthesis of behaviours that promote co-worker relationships in long-term residential care. By identifying the specific behaviours that underpin capability, opportunity and motivation, and by demonstrating the central role of leadership in shaping relational environments, the review complements cultural descriptions by adding concrete modifiable behaviours. These insights form the basis for workforce development, leadership training and intervention research aimed at strengthening supportive teams in long-term residential care.

## CRediT authorship contribution statement

**Kirsty Haunch:** Writing – original draft, Validation, Software, Resources, Project administration, Methodology, Investigation, Funding acquisition, Formal analysis, Data curation, Conceptualization. **Kirstine McDermid:** Resources, Data curation. **Karen Spilsbury:** Validation, Supervision, Methodology, Funding acquisition, Conceptualization.

## Declaration of competing interest

The authors declare that they have no known competing financial interests or personal relationships that could have appeared to influence the work reported in this paper.

## Appendix Supplementary materials

Image, application 1

Image, application 2

Image, application 3

Image, application 4
