# Supplementary material for: Staff behaviours that promote positive co-worker relationships in health and social care settings: A scoping review with implications for long-term residential care
Source: Int J Nurs Stud Adv. 2026 Apr 19;10:100543. doi: 10.1016/j.ijnsa.2026.100543 (PMC13101673; doi:10.1016/j.ijnsa.2026.100543)
Supplement: Supplementary file 2 [file mmc2.docx]

**Supplementary File 5: Full Search Strategy and Update Summary**

**Purpose**

This file provides the complete search strategies used for the scoping review, including exact search strings, Boolean logic, and grey literature sources consulted. It also documents the updated search conducted in October 2025 and explains why newly identified studies were not incorporated into the main synthesis. This information is provided to support transparency and enable future replication.

**1. Original Search Strategy**

**Date of Original Search:** June 2024
**Databases Searched:**

- Ovid MEDLINE
- CINAHL
- PsycINFO
- EMBASE
- Cochrane Library
- Web of Science
- Scopus

**Grey Literature Sources:**

- Agency for Healthcare Research and Quality (AHRQ)
- American Nurses Association (ANA)
- Academy of Management
- My Home Life
- Care Quality Commission (CQC)
- Skills for Care

**Search Approach:**
The search strategy was designed to capture literature on co-worker relationships, workplace culture, and quality of care in health and social care settings. It combined controlled vocabulary (e.g., MeSH, Emtree) and free-text terms using Boolean operators and adjacency rules.

**Example: Ovid MEDLINE Search Strategy**

Database: Ovid MEDLINE(R) <1946 to August Week 2 2020>

Search Strategy:

1 Quality Assurance, Health Care/

2 Quality Indicators, Health Care/

3 Outcome Assessment, Health Care/

4 treatment outcome/ or treatment failure/

5 Total Quality Management/

6 Quality Improvement/

7 "Standard of Care"/

8 "Quality of Health Care"/

9 (quality of health care or quality of healthcare or quality care or care quality).tw.

10 "standard* of care".tw.

11 (quality adj2 (improv* or assurance or change)).tw.

12 ((care or healthcare) adj2 (improv* or change)).tw.

13 Patient Safety/

14 patient safety.tw.

15 exp Medication Errors/

16 medication error*.tw.

17 mortality/ or hospital mortality/

18 Morbidity/

19 Patient Satisfaction/

20 patient satisfaction*.tw.

21 (patient adj1 death*).tw.

22 or/1-21 [quality or outcome]

23 (worker* adj2 (relations or relationship*)).tw.

24 ((employee* or colleague*) adj2 (relations or relationship*)).tw.

25 (staff adj relations).tw.

26 (staff adj1 relationships).tw.

27 (workplace adj (relations or relationship*)).tw.

28 ((supervisor or supervisors) adj2 (relations or relationship*)).tw.

29 management relations.tw.

30 ("relationship* with managers" or "relationship with management").tw.

31 (subordinate* adj2 (relations or relationship*)).tw.

32 (interprofessional adj1 (relations or relationship*)).tw.

33 *organizational culture/

34 employee grievances/

35 personnel loyalty/

36 ((work* or workplace) adj2 culture).tw.

37 ((work* or workplace) adj2 bullying).tw.

38 ((work or workplace) adj2 conflict).tw.

39 organi?ational social capital.tw.

40 social capital/

41 ((nurse* or care home assistant*) adj2 relationship*).tw.

42 work environment.ti.

43 work environment.ab. /freq=2

44 psychosocial environment.tw.

45 or/23-44 [workplace culture]

46 Primary Health Care/

47 exp General Practice/

48 general practitioners/ or physicians, family/ or physicians, primary care/

49 Ambulatory Care/

50 community pharmacy services/

51 community health services/ or community health nursing/ or home health nursing/ or home care services/ or home nursing/

52 community mental health services/ or mental health services/

53 community pharmacy services/

54 maternal health services/

55 emergency medical services/ or emergency service, hospital/ or trauma centers/ or emergency services, psychiatric/

56 exp nursing care/ or nursing services/

57 Homes for the Aged/ or "home* for the aged".tw.

58 exp Nursing Homes/ or nursing home*.tw.

59 "care home*".tw.

60 Long-Term Care/

61 ((long-term or longterm or long-stay or longstay or retir*) adj5 (facilit* or institution* or setting* or resident*)).tw.

62 Residential Facilities/

63 (gp* or general practitioner* or family doctor* or family practitioner* or family physician* or local doctor* or primary care doctor* or primary care physician*).tw.

64 (general practice* or family practice* or family medicine).tw.

65 exp Hospitals/

66 exp Nursing Staff/

67 (primary care or secondary care).tw.

68 or/46-67 [setting]

69 22 and 45 and 68

70 limit 69 to (english language and yr="1990 -Current")

**Other Databases:**
Equivalent strategies were adapted for CINAHL, PsycINFO, EMBASE, Cochrane Library, Web of Science, and Scopus.
*(See original file for full details.)*

**2. Updated Search**

**Date of Update:** October 2025
**Databases and Sources:** Same as original search
**Search Parameters:** Extended date range to include literature published up to October 2025

**Results:**

- **New Papers Identified:**
  - Varga et al., 2025
  - Zettna et al., 2025
- **Summary of Findings:**
  Both papers reinforced existing themes reported in the scoping review, including the importance of interpersonal dynamics and leadership behaviours. No new themes emerged.
- **Decision:**
  These studies were not integrated into the main synthesis because they did not alter conclusions. This approach aligns with guidance on search currency (Stokes, Sutcliffe & Thomas, 2023).

**3. Rationale for Exclusion**

The updated search identified two studies that supported existing findings but introduced no new concepts. Integrating these studies would not have changed the interpretation or conclusions of the review. To maintain transparency, these studies are documented here rather than incorporated into the main report.

### ****4. Replication Guidance****

All search strings, grey literature sources, and update details are provided to enable replication of the review process.
