# Supplementary material for: Staff behaviours that promote positive co-worker relationships in health and social care settings: A scoping review with implications for long-term residential care
Source: Int J Nurs Stud Adv. 2026 Apr 19;10:100543. doi: 10.1016/j.ijnsa.2026.100543 (PMC13101673; doi:10.1016/j.ijnsa.2026.100543)
Supplement: Supplementary file 3 [file mmc3.docx]

| Supplementary Table 2  **Quality assessment of included studies using MMAT for primary studies and AMSTAR 2 for secondary reviews of literature** | | | | | | | | | | | | | |
| --- | --- | --- | --- | --- | --- | --- | --- | --- | --- | --- | --- | --- | --- |
| Qualitative  Included studies | | **Quality checklist criteria for all types of study** | | | | | | | | | | | |
|  |  | Screening questions | | | Qualitative studies | | | | | | | | |
|  | | Are there clear research questions/aims? | | Do the collected data allow to address the research questions/aims? | Is the qualitative approach appropriate to answer the research question/aim? | | Are the qualitative data collection methods adequate to address the research question/aim? | | Are the findings adequately derived from the data? | | Is the interpretation of results sufficiently substantiated by data? | | Is there coherence between qualitative data sources, collection, analysis and interpretation? |
| Hoare et al (2013) Constructivist Grounded Theory  Overall judgement: Methodologically sound qualitative study, with strong coherence between methodology, data collection, analysis and interpretation. | | Yes  The study clearly aims to explore how practice nurses use information and how reciprocal role modelling develops between graduate and experienced practice nurses in general practice. | | Yes  Data from observations, field notes, and in‑depth interviews with graduate and experienced nurses directly address the study aims. | Yes  A constructivist grounded theory approach is well suited to exploring social processes and relational dynamics such as role modelling. | | Yes  The use of ethnographic observation, unstructured interviews, and theoretical sampling is appropriate and consistent with grounded theory methodology. | | Yes  Categories and sub‑categories are clearly grounded in participant quotations, memos, and observational data. | | Yes  Interpretations are well supported by rich data extracts, analytic memos, and a transparent explanation of theory development. | | Yes  There is strong coherence between the data sources, grounded theory analytic procedures, and the resulting theoretical model. |
| Kim and Oh (2016)  Grounded Theory  Overall judgement: Methodologically sound qualitative study with strong theoretical coherence and transparent analytic procedures. | | Yes  The study clearly aims to develop a substantive theory explaining communication processes among clinical nurses within hierarchical organisational cultures in Korea. | | Yes  In‑depth interviews with nurses of varying experience levels provide data well aligned to exploring communication processes and organisational culture. | Yes  A grounded theory approach is appropriate for examining social processes and generating explanatory models of communication behaviour. | | Yes  Use of prolonged, in‑depth interviews, theoretical sampling, and iterative data collection is consistent with grounded theory methodology. | | Yes  Analytic categories and phases are clearly developed from systematic coding and supported by extensive participant quotations. | | Yes  Interpretations are supported by rich data extracts and transparent links between data, categories, and the resulting theory | | Yes  There is strong coherence between data collection, grounded theory analytic procedures, and the constructed explanatory framework. |
| Duddle et al (2007)  Multiple case study  Overall judgement: Methodologically sound qualitative case study, with coherent alignment between design, data collection, analysis and interpretation. | | Yes  The study clearly aims to explore how registered nurses relate to and interact with each other in the workplace and to identify factors influencing intra-professional interactions. | | Yes  Interview data, supported by field notes and contextual information across multiple wards, are well aligned with exploring nurses’ workplace interactions. | Yes  A multiple case study design is appropriate for examining intra-professional relationships within their real‑world clinical context. | | Yes  In‑depth semi‑structured interviews, supplemented by informal discussions and field notes, are appropriate to capture relational and interactional experiences. | | Yes  Thematic analysis is clearly explained and findings are grounded in participants’ accounts, with illustrative excerpts used to support themes. | | Yes  Interpretations are supported by rich narrative data and linked to existing literature, with careful attention to participants’ perspectives. | | Yes  There is good coherence between the study aim, case study design, data collection methods, analytic approach, and thematic interpretation. |
| Padgett et al (2013)  Ethnographic case study  Overall judgement: Methodologically sound ethnographic study, with strong coherence between design, data sources, analysis and interpretation. | | Yes  The study clearly aims to examine how staff nurses negotiate collegiality, accountability, and peer monitoring in everyday practice. | | Yes  Data from participant observation, semi‑structured interviews, and policy analysis directly address the study aims. | Yes  An ethnographic approach is appropriate for exploring everyday practices, interactions, and organisational contexts shaping collegial behaviour. | | Yes  Prolonged participant observation, interviews, and documentary analysis provide rich and appropriate data for the research focus. | | Yes  Findings are systematically derived from observational and interview data, with analytic categories clearly grounded in participants’ accounts. | | Yes  Interpretations are well supported by detailed empirical examples and coherent analytic reasoning. | | Yes  There is strong coherence between the study’s ethnographic design, data collection methods, analytic approach, and explanatory conclusions. |
| Madden et al (2017) Grounded theory study  Overall judgement: Methodologically sound qualitative grounded theory study, with clear analytic rigor and coherence between data, analysis, and interpretation. | | Yes  The study clearly aims to develop a theoretical understanding of nurse–CNA communication processes during direct care in nursing home settings. | | Yes  Data from observation, shadowing, and semi‑structured interviews with nurses and CNAs directly address the study aims. | Yes  A grounded theory approach is appropriate for examining socially constructed communication processes within a complex care environment | | Yes  Use of multiple data sources (observation, shadowing, interviews) and theoretical sampling is appropriate and methodologically robust. | | Yes  The four “rules of performance” are clearly derived through constant comparative analysis and grounded in participants’ accounts. | | Yes  Interpretations are supported by rich empirical examples and transparent analytic reasoning. | | Yes  There is strong coherence between the grounded theory design, data collection methods, analytic process, and resulting conceptual framework. |
| Lux et al (2014) Qualitative descriptive study  Overall judgement: Methodologically sound qualitative descriptive study, with clear alignment between study aims, data collection, analysis, and interpretation. | | Yes  The study clearly aims to identify staff nurses’ recommendations for nursing education strategies to prepare new graduates to manage disruptive behaviour in the workplace. | | Yes  Individual semi‑structured interviews with experienced staff nurses directly address the study aims. | Yes  A qualitative descriptive approach is appropriate for eliciting practice‑based recommendations and experiential perspectives. | | Yes  One‑to‑one semi‑structured interviews are suitable for exploring nurses’ views on education, communication, and workplace culture. | | Yes  Themes are derived through systematic content analysis with transparent coding and categorisation processes. | | Yes  Interpretations are supported by representative participant quotations and linked clearly to the identified themes. | | Yes  There is good coherence between the study aims, data collection methods, analytic approach, and resulting interpretations. |
| Schirm et al (2000) Qualitative focus‑group study  Overall judgement: Methodologically sound qualitative focus‑group study, with coherent alignment between aims, data collection, analysis, and interpretation. | | Yes  The study clearly aims to explore licensed nurses’ and nursing assistants’ perceptions of good caregiving and factors that facilitate or hinder care delivery in nursing homes. | | Yes  Data from multiple focus groups with licensed nurses and nursing assistants directly address perceptions of caregiving roles, teamwork, and barriers to quality care. | Yes  A qualitative focus‑group approach is appropriate for exploring shared and divergent perceptions among different staff groups within nursing homes. | | Yes  Separate focus groups, parallel and divergent questioning, and verbatim transcription provide appropriate depth and comparative insight. | | Yes  Themes are systematically developed through content analysis and supported by extensive participant quotations. | | Yes  Interpretations are clearly grounded in participants’ accounts and linked to role theory and organisational context. | | Yes  There is strong coherence between study aims, focus‑group design, analytic approach, and the resulting thematic interpretation. |
| Munkejord et al (2019) Constructivist grounded theory study  Overall judgement: Methodologically sound qualitative grounded theory study, with strong coherence between study aims, data collection, analysis, and interpretation. | | Yes  The study clearly aims to examine how healthcare managers can promote connection and ethnic equality among staff in multicultural nursing home organisations. | | Yes  In‑depth interviews with all staff members in a strategically selected nursing home unit provide data well aligned with the study aims. | Yes  A constructivist grounded theory approach is appropriate for exploring social processes, hierarchies, and inclusion within a multicultural care setting. | | Yes  Use of open‑ended, in‑depth interviews with staff across roles and backgrounds is appropriate and allows rich exploration of everyday work practices. | | Yes  The three “golden rules” and associated organisational measures are clearly derived through systematic coding and constant comparison. | | Yes  Interpretations are well supported by detailed participant accounts and illustrative quotations. | | Yes  There is strong coherence between the grounded theory design, data collection, analytic process, and the resulting conceptual framework. |
| Potter et al (2004)  Qualitative descriptive study (focus‑group based)  Overall judgement: Methodologically sound qualitative descriptive study, with clear coherence between aims, data collection, analysis, and interpretation. | | Yes  The study clearly aims to examine RN–unlicensed assistive personnel (UAP) working relationships and the care delivery practices that influence collaboration and patient care outcomes. | | Yes  Focus‑group data from RNs and UAPs across multiple patient care units directly address perceptions of working relationships and care delivery practices. | Yes  A qualitative descriptive approach using focus sessions is appropriate for exploring relational dynamics and workplace practices. | | Yes  Separate focus groups for RNs and UAPs enable comparison of perspectives and reduce role‑related power constraints in discussion. | | Yes  Themes such as trust, initiative, communication, assignment patterns, and “knowing the patient” are clearly grounded in participants’ accounts. | | Yes  Interpretations are well supported by illustrative quotations and are logically linked to identified care delivery practices. | | Yes  There is strong coherence between study aims, focus‑group design, thematic analysis, and practice‑oriented conclusions. |
| Jakobsen et al (2018)  Qualitative focus‑group study  Overall judgement: Methodologically sound qualitative focus‑group study, with clear alignment between aims, data collection, analysis, and interpretation. | | Yes  The study clearly aims to identify barriers and facilitators of collaboration among eldercare workers and to describe processes supporting well‑functioning collaboration. | | Yes  Focus‑group data from eldercare workers across seven care homes directly address everyday collaboration practices and challenges. | Yes  An exploratory qualitative approach using focus groups is appropriate for examining relational, organisational, and contextual aspects of collaboration. | | Yes  Use of facilitated focus groups with workers across roles, units, and shifts provides rich, comparative data on collaboration. | | Yes  Themes describing barriers, facilitators, and supporting processes are systematically derived through iterative coding and cross‑group comparison. | | Yes  Interpretations are well supported by detailed participant quotations and are clearly linked to observed collaboration practices. | | Yes  There is strong coherence between study aims, focus‑group design, analytic strategy, and the resulting thematic framework. |
| Norikoshi et al (2017)  Qualitative interview study  Overall judgement: Methodologically sound qualitative study, with coherent alignment between aims, data collection, analytic approach, and interpretation. | | Yes  The study clearly aims to identify the attributes of nurses’ workplace social capital in Japan. | | Yes  Semi‑structured interviews with nurses across seven hospitals directly address perceptions of workplace social capital and related organisational attributes. | Yes  A qualitative approach is appropriate for exploring socially constructed attributes of workplace relationships and social capital. | | Yes  Individual semi‑structured interviews allow in‑depth exploration of nurses’ experiences and perceptions. | | Yes  The six attributes of workplace social capital are systematically derived through structured application of the Kawakita Jiro (KJ) method. | | Yes  Interpretations are supported by participant quotations and transparent analytic procedures. | | Yes  There is strong coherence between the study aim, interview data, KJ analytic process, and resulting conceptual categorisation. |
| Bellury et al (2016) Qualitative descriptive study (focus groups and open‑ended survey responses)  Overall judgement: Methodologically sound qualitative descriptive study, offering coherent and well‑supported insights into divergent perceptions of teamwork among acute care staff. | | Yes  The study clearly aims to explore perceptions of teamwork among nursing assistive personnel (NAP) and registered nurses (RNs) in acute care settings and to compare perspectives between the two groups. | | Yes  Focus‑group data from NAP and open‑ended survey responses from RNs directly address experiences and perceptions of teamwork and coordination. | Yes  A qualitative descriptive approach is appropriate for capturing and comparing perceptions of teamwork across occupational groups. | | Yes  Use of NAP focus groups and open‑ended RN survey responses allows inclusion of both perspectives, though with differing depths of data generation. | | Yes  Themes related to shared mental models, closed‑loop communication, and mutual trust are systematically derived through inductive analysis and mapped to an established teamwork framework. | | Yes  Interpretations are supported by rich illustrative quotations from NAP and comparative examples from RN responses. | | Yes  There is strong coherence between the study aims, qualitative design, analytic approach, and the resulting interpretive framework. |
| Ericson-Lidman et al (2015) Participatory action research study  Overall judgement: Methodologically sound qualitative participatory action research study, with strong alignment between aims, collaborative design, analytic approach, and interpretation. | | Yes  The study clearly aims to describe a participatory action research intervention process enabling care providers to deal constructively with troubled conscience related to perceptions of deficient teamwork. | | Yes  Data from repeated PAR sessions, supported by recorded group discussions and reflective processes, directly address experiences of troubled conscience and teamwork. | Yes  Participatory action research is appropriate for exploring lived experiences, collective reflection, and practice change related to teamwork and moral distress. | | Yes  Use of longitudinal, facilitated PAR sessions with audio‑recording allows in‑depth exploration of perceptions, learning processes, and action development over time. | | Yes  Domains describing problem identification, understanding, action, and evaluation are systematically derived through domain analysis of PAR session transcripts. | | Yes  Interpretations are supported by participant quotations and transparent linkage between reflections, actions, and emerging understanding. | | Yes  There is strong coherence between the PAR design, reflective group processes, analytic framework, and the reported learning and practice implications. |
| Colon-Emeric et al (2014) Qualitative evaluation using focus groups  Overall judgement: Methodologically robust qualitative study with clear aims, appropriate design, rigorous analysis, and coherent interpretation. | | Yes  The study clearly aims to examine whether staff descriptions of learning climate, learning processes, and perceived outcomes differ between nursing homes receiving CONNECT + FALLS versus FALLS alone. | | Yes  Focus group data directly capture staff perceptions of learning, communication, teamwork, and outcomes, which align with the stated research aims. | Yes  A qualitative approach is appropriate for exploring learning processes, social interactions, and organisational mechanisms underlying intervention uptake. | | Yes  Multiple focus groups across intervention and control facilities, guided by a structured interview protocol, provide rich and relevant data. | | Yes  Findings are systematically derived using framework analysis, with transparent coding, theme development, and comparison between groups. | | Yes  Interpretations are supported by extensive participant quotations and consistent thematic patterns across facilities and staff roles. | | Yes  There is strong coherence between the study aims, theoretical framework (social constructivist learning), data collection, analytic approach, and interpretation. |
| Quantitative  Included studies | | Screening questions | | | Quantitative studies | | | | | | | | |
|  | | Are there clear research questions/aims? | | Do the collected data allow to address the research questions/aims? | Is the sampling strategy relevant to address the research question/aim? | | Is the sample representative of the target population? | | Are the measurements appropriate? | | Is the risk of non-response bias low? | | Is the statistical analysis appropriate to answer the research question? (or study aim?) |
| Jakobsen et al (2020)  Overall judgement  Methodologically sound overall, with limitations relating to attrition and potential non‑response bias that support cautious interpretation of findings. | | Yes  The aim and hypotheses are clearly stated (effect of participatory intervention on social capital and organizational readiness for change) | | Yes  Repeated measures of validated social capital and readiness scales collected at baseline, 6 and 12 months. | Yes  Cluster randomization at department level is clearly described  Allocation was concealed  Randomization conducted by a person blinded to department status  Appropriate choice to reduce contamination | | Yes  Baseline characteristics reported  No statistically significant differences between intervention and control groups at baseline  Similar demographic profiles | | Can’t tell  Blinding was not possible due to the intervention; outcomes were self‑reported and analyst blinding was not explicitly reported.  Outcomes are self‑reported questionnaires, which increases risk of performance and detection bias  It is not explicitly stated whether analysts were blinded during outcome assessment | | Can’t tell  Substantial loss to follow‑up (>50% at 12 months) means non‑response bias cannot be ruled out, despite appropriate statistical handling of missing data. | | Yes  Mixed‑effects models appropriately accounted for clustering, repeated measures, and missing data, and were suitable for the study design and aims. |
| Thomas et al (2013) Quantitative quality‑improvement before–after study  Overall judgement: The study meets MMAT criteria for quantitative research, with appropriate aims, measures, and analyses, though limited by absence of a control group and incomplete reporting of response rates. | | Yes  The study clearly aims to evaluate whether implementation of TeamSTEPPS improves patient safety culture and teamwork across a healthcare system. | | Yes  Pre‑ and post‑implementation safety culture survey data (HSOPSC) are directly relevant to assessing changes in safety culture and teamwork perceptions. | Yes  Inclusion of multidisciplinary staff across multiple hospitals and care settings is appropriate for assessing organisational‑level safety culture change. | | Yes  The sample includes a broad cross‑section of healthcare staff within the health system; however, representativeness beyond the single health system is limited. | | Yes  The AHRQ Hospital Survey on Patient Safety Culture is a validated and widely used instrument for assessing teamwork and safety culture. | | Can’t tell  Response rates are not consistently reported across all survey administrations, limiting assessment of non‑response bias. | | Yes  Descriptive and comparative analyses of pre‑ and post‑intervention survey scores are appropriate for evaluating changes in safety culture over time. |
| Kalisch et al (2007)  Overall judgement: The study meets MMAT criteria for quantitative non‑randomised studies, with clear aims, appropriate measures, and suitable analyses, but is limited by single‑site design and lack of a control group. | | Yes  The study clearly aims to determine the impact of a teamwork and staff‑engagement intervention on patient fall rates, staff assessments of teamwork, patient satisfaction, and staff turnover and vacancy rates. | | Yes  Pre‑ and post‑intervention data on falls, staff turnover/vacancy, patient satisfaction, and staff ratings of teamwork directly address the stated aims. | Yes  Inclusion of all nursing staff on a single medical oncology unit is relevant for evaluating a unit‑level teamwork intervention. | | Yes  The sample is limited to one hospital unit in a single organisation, which limits representativeness of wider nursing populations. | | Yes  Objective outcomes (fall rates, turnover, vacancy rates) and validated instruments are appropriate; staff teamwork perceptions are measured using structured interviews. | | Yes  Staff participation was very high (97%), reducing the likelihood of non‑response bias for staff‑reported outcomes. | | Yes  Pre‑ and post‑intervention comparisons using appropriate statistical tests (e.g. t‑tests, χ² tests) are suitable for assessing change over time within the unit. |
| DeMiglo et al (2005) Quasi‑experimental pre–post team‑building intervention study  Overall judgement: The study meets MMAT criteria for quantitative non‑randomised research, with clear aims, appropriate measures, and suitable analyses, but is limited by single‑site design and potential non‑response bias. | | Yes  The study clearly aims to determine the impact of a team‑building intervention on group cohesion, nurse satisfaction, and nurse turnover rates. | | Yes  Pre‑ and post‑intervention data on group cohesion, nurse satisfaction, and turnover directly address the stated aims. | Yes  Inclusion of registered nurses working on inpatient units within the hospital is appropriate for evaluating a unit‑based team‑building intervention. | | Yes  The sample is drawn from a single Magnet‑designated teaching hospital, limiting representativeness of the wider nursing workforce. | | Yes  Validated and reliable instruments were used (Group Cohesion Scale, NDNQI Adapted Index of Work Satisfaction and Job Enjoyment), alongside objective turnover data. | | Yes  Survey response rates were moderate to low (47% pre‑intervention; 34% post‑intervention), indicating a potential risk of non‑response bias. | | Yes  Descriptive statistics and appropriate non‑parametric tests (Mann–Whitney Rank Sum) were used to compare pre‑ and post‑intervention outcomes. |
| Heponiemi et al (2011) Cross‑sectional survey of team climate by ownership type  Overall judgement: The study meets MMAT criteria for quantitative non‑randomised research, with clear aims, appropriate measures, and robust statistical analyses, though generalisability is limited to female staff in the Finnish elderly care context. | | Yes  The study clearly aims to examine the association between ownership type and perceived team climate in elderly care facilities, and to test whether work stress factors mediate or moderate this association. | | Yes  Survey data on ownership type, team climate dimensions, and work stress factors directly address the stated aims. | Yes  Recruitment of older people care staff from multiple elderly care facilities with different ownership types is appropriate for comparing team climate across organisational forms. | | Partly  The large national sample strengthens representativeness; however, inclusion was limited to Finnish female care staff, limiting generalisability to male staff or other countries. | | Yes  Validated instruments were used (Team Climate Inventory, established stress scales with good reliability), and ownership type was clearly categorised. | | Yes  The response rate was relatively high (66%), reducing the likelihood of substantial non‑response bias. | | Yes  Analyses of covariance and interaction testing were appropriate for examining associations and moderating effects while controlling for relevant confounders. |
| Barry et al (2019) Cross‑sectional survey  Overall judgement: The study meets MMAT criteria for quantitative non‑randomised research, with clear aims, appropriate measures, and suitable analyses, though generalisability is limited by sample size and setting. | | Yes  The study clearly aims to examine nursing staff perceptions of inclusion as care‑team members and to assess whether perceived inclusion is associated with overall empowerment and its specific dimensions. | | Yes  Survey data on perceived inclusion by different team members and validated empowerment measures directly address the stated research aims. | Yes  Inclusion of licensed nurses and nurse aides working in long‑term care facilities is appropriate for examining team inclusion and empowerment within this care context. | | Yes  The sample was drawn from four facilities and included a relatively small number of participants (n = 95), limiting representativeness of the wider long‑term care nursing workforce. | | Yes  The study used established and validated instruments, including the Perception of Empowerment Instrument, and clearly defined measures of team inclusion. | | Yes  The response rate was relatively high (approximately 70%), suggesting a low risk of non‑response bias. | | Yes  Correlation analyses and multiple linear regression models were appropriate for examining associations between perceived inclusion and empowerment |
| Brunetto et al (2013) Cross‑sectional survey of supervisor–nurse relationships, teamwork, wellbeing and turnover | | Yes  The study clearly aims to examine relationships between supervisor–nurse relationships, teamwork, psychological wellbeing, affective commitment, and turnover intentions among North American nurses, including differences across generational cohorts. | | Yes  Survey data measuring supervisor–subordinate relationships, teamwork, wellbeing, affective commitment, and turnover intentions directly address the stated research aims. | Yes  Recruiting registered nurses from two comparable acute care hospitals is appropriate for examining workplace relationships, wellbeing, and retention intentions in a North American hospital context. | | Partly  The large sample size (n = 730) strengthens internal validity; however, data were drawn from two private-sector hospitals, limiting generalisability to public or not-for-profit hospitals or other national contexts. | | Yes  Established and validated instruments were used (e.g. LMX-7 scale, teamwork scale, affective commitment scale, wellbeing scale, turnover intention scale), with strong reported reliability. | | Partly  The response rate was moderate (approximately 40%), which is acceptable for organisational surveys but suggests some potential for non-response bias. | | Yes  Correlation analysis, multiple regression analyses, and MANOVA were appropriate for testing relationships between variables and examining differences across generational cohorts. |
| Winning et al (2017) Cross‑sectional survey of errors/adverse events, emotional distress, and co-worker support in NICU staff  Overall judgement: The study meets MMAT criteria for quantitative non‑randomised research, with clear aims, appropriate measures, and statistical analyses, though representativeness is limited by setting, sample composition, and response rate. | | Yes  The study clearly aims to examine the emotional and professional impact of errors or adverse events on NICU healthcare providers and to assess whether co-worker support moderates these associations. | | Yes  Survey data capturing experiences of errors/adverse events, emotional distress (anxiety, depression), professional quality of life, and perceived co-worker support directly address the stated aims. | Yes  Inclusion of all healthcare providers involved in patient care across multiple neonatal intensive care units is appropriate for examining the impact of adverse events and co-worker support in this high‑risk clinical context. | | Partly  The large sample size (n = 463) strengthens internal validity; however, participants were drawn from a single paediatric hospital system and were predominantly white and female, limiting broader generalisability. | | Yes  Validated and reliable instruments were used, including the Hospital Anxiety and Depression Scale (HADS), Professional Quality of Life Scale (ProQOL), and Survey of Perceived Co-worker Support (SPCS), with strong reported reliability. | | Partly  The response rate was moderate (approximately 46%), which is acceptable for workplace surveys but indicates some potential for non‑response bias. | | Group comparisons (ANOVA, chi‑square) and hierarchical linear regression analyses were appropriate for testing differences between exposure groups and examining moderating effects of co-worker support. |
| Lehmann-Willenbrock et al (2012) Cross‑sectional survey of age diversity appreciation, trust, and nurse well‑being  Overall judgement: The study meets MMAT criteria for quantitative non‑randomised research, with clear aims, appropriate measures, and robust analytic methods, though generalisability is limited by single‑site design and sample size. | | Yes  The study clearly aims to examine whether appreciation of age diversity is associated with nurses’ well‑being (stress and work–life balance) and team commitment, and whether trust in co‑workers mediates these relationships. | | Yes  Survey data measuring age diversity appreciation, co‑worker trust, stress, work–life balance, and team commitment directly address the stated aims. | Yes  Recruiting nurses from multiple departments within a large German hospital is appropriate for examining age diversity perceptions and their relationship with well‑being and commitment in nursing teams. | | Partly  The sample reflects the demographic profile of the hospital’s nursing workforce; however, it is limited to a single hospital and a modest sample size (n = 138), restricting wider generalisability. | | Yes  Validated and reliable German‑language instruments were used to assess age diversity appreciation, co‑worker trust, stress, work–life balance, and team commitment, with strong reported internal consistency. | | Yes  The response rate was relatively high (approximately 65%), reducing the likelihood of substantial non‑response bias. | | Yes  Correlational analyses and hierarchical regression models with mediation testing (Baron & Kenny approach and Sobel tests) were appropriate for examining the hypothesised relationships and mediation effects. |
| Quoidbach et al (2009) Cross‑sectional team‑level study of emotional intelligence, team performance, and cohesion in nursing teams | | Yes  The study clearly aims to examine the relationships between trait emotional intelligence, nursing team performance (across multiple dimensions), and team cohesiveness in real‑world nursing teams. | | Yes  Team‑level data on emotional intelligence, cohesiveness, and multiple indicators of performance (job satisfaction, supervisor ratings, turnover, and health‑care quality) directly address the stated aims. | Yes  Inclusion of all members from 23 nursing teams within a hospital setting is appropriate for examining team‑level emotional intelligence, cohesion, and performance. | | Partly  The study involved real clinical nursing teams, strengthening ecological validity; however, the number of teams was relatively small (n = 23), and all were drawn from a single hospital, limiting generalisability. | | Yes  Established and theoretically grounded instruments were used, including a validated trait emotional intelligence scale, a recognised group cohesiveness scale, and multi‑source performance indicators | | Yes  All members of each nursing team participated, reducing the risk of non‑response bias at the team level, although anonymous data collection limited assessment of individual characteristics. | | Yes  Correlational analyses at the team level were appropriate for examining relationships between emotional intelligence dimensions, cohesiveness, and distinct aspects of team performance. |
| Kalisch et al (2010) Psychometric development and testing of the Nursing Teamwork Survey  Overall judgement: The study meets MMAT criteria for quantitative non‑randomised research, with clear aims, appropriate sampling and measures, and robust statistical analyses, though external generalisability is limited by the small number of sites. | | Yes  The study clearly aims to develop and test the psychometric properties (reliability and validity) of the Nursing Teamwork Survey (NTS) for use with inpatient nursing teams in acute care settings. | | Yes  Large‑scale survey data were specifically collected to evaluate content, construct, concurrent, convergent, and contrast validity, as well as reliability of the instrument. | Yes  Inclusion of nursing staff from 38 inpatient units across two hospitals is appropriate for testing an instrument intended for unit‑level measurement of nursing teamwork. | | Partly  The large sample (n = 1,758) and inclusion of multiple inpatient unit types strengthen representativeness; however, data were drawn from only two hospitals, limiting broader generalisability. | | Yes  The Nursing Teamwork Survey was theoretically grounded (Salas “Big Five” framework), developed via literature review and focus groups, and tested against validated comparison instruments (e.g. Safety Attitudes Questionnaire). | | Partly  The response rate was moderate (56.9%), which is acceptable for organisational surveys, but some potential for non‑response bias remains. | | Yes  The use of exploratory and confirmatory factor analysis, intra class correlations, test–retest reliability, internal consistency testing, and multiple forms of validity testing was appropriate and rigorous for psychometric evaluation. |
| Yan et al (2022) Cross‑sectional study of social support, psychological resilience, and quality of life among nurses in infectious disease departments  **Overall judgement:** The study meets MMAT criteria with clear aims, appropriate and reliable measures, and strong analytic methods; however, causal inference and broader generalisability are limited by the cross‑sectional design and convenience sampling. | | Yes  The study clearly aims to examine relationships between social support and quality of life among nurses in infectious disease departments, and to test whether psychological resilience mediates this relationship. | | Yes  Survey data measuring social support, psychological resilience, and quality of life directly address the stated aims, and mediation was explicitly tested using structural equation modelling. | Yes  Recruiting nurses from infectious disease departments across ten general hospitals in different regions of China is appropriate for examining these psychosocial relationships in this specific workforce | | **Partly**  The large sample size (n = 845) strengthens internal validity; however, convenience sampling and restriction to Chinese general hospitals limit generalisability to other healthcare systems or cultural contexts. | | Yes  Well‑established and validated instruments were used, including the Social Support Rating Scale, Connor–Davidson Resilience Scale, and WHOQOL‑BREF, all demonstrating good internal consistency. | | Yes  The questionnaire recovery rate was very high (97.6%), suggesting a low risk of non‑response bias. | | Yes  Pearson correlations and structural equation modelling with bootstrap mediation testing were appropriate and robust methods for examining direct and indirect relationships among variables. |
| Kaya et al (2022) Cross‑sectional study  Overall judgement: The study meets MMAT criteria for quantitative non‑randomised research, with clear aims, appropriate measures, and suitable analyses; however, interpretation is limited by low response rate and restricted sampling frame. | | Yes  The study clearly aims to determine nurses’ positive psychological capital levels and to examine the relationships between positive psychological capital, employee voice, and organizational silence behaviours. | | Yes  Survey data measuring positive psychological capital, employee voice, and organizational silence directly address the stated aims. | Yes  Recruiting nurses from both a public and a private university hospital is appropriate for examining psychological capital and communication‑related behaviours within nursing work environments. | | Partly  The sample includes nurses from two different hospital types; however, the participation rate was relatively low (36.7%), and sampling was limited to two hospitals in Turkey, restricting generalisability. | | Yes  Validated and culturally adapted instruments were used, including the Positive Psychological Capital Scale, Employee Voice Scale, and Organizational Silence Scale, with good reported internal consistency. | | No  The moderate response rate (36.7%) suggests a potential risk of non‑response bias. | | Yes  Descriptive statistics, group comparisons, and Pearson correlation analyses were appropriate for examining relationships among the study variables. |
| Phan et al (2022) Educational intervention using simulation and cognitive rehearsal to address bullying in new graduate nurses  Overall judgement: The study meets MMAT criteria for quantitative non‑randomised research, with clear aims, appropriate intervention design, valid outcome measures, and high participation rates; however, conclusions are constrained by small sample size, single‑site implementation, lack of a control group, and reliance on self‑reported outcomes. | | Yes  The study clearly aims to enhance new graduate nurses’ ability to identify and respond to workplace bullying through an educational intervention using simulation role‑play and cognitive rehearsal | | Yes  Pre‑intervention and multiple post‑intervention measurements collected using the Clark Workplace Civility Index and Kirkpatrick evaluation levels directly address the study aims. | Yes  Recruiting new graduate nurses enrolled in a nurse residency programme is appropriate for evaluating an intervention targeting early‑career nurses at high risk of workplace bullying. | | Partly  The sample consisted of a single cohort of new graduate nurses from one large academic health system (n = 36), which is suitable for feasibility testing but limits generalisability to other settings and populations. | | Yes  The study employed validated and widely used tools, including the Clark Workplace Civility Index and Kirkpatrick’s training evaluation framework, which are appropriate for assessing perceived civility, learning, behaviour change, and practice impact. | | Yes  Response rates were high across data collection points (86–100%), reducing the likelihood of non‑response bias, although self‑reported measures may still be influenced by social desirability. | | Yes  Descriptive statistics and repeated‑measures analysis of variance were appropriate for examining changes in civility scores over time; qualitative analysis of open‑ended responses appropriately complemented quantitative findings |
| Pedersen et al (2023) Cross‑sectional study | | Yes  The study clearly aims to examine the associations between different subtypes of workplace social capital (bonding, bridging, and linking) and self‑reported quality of health care services, and to compare these effects with workload and work pace. | | Yes  Large‑scale questionnaire data measuring social capital, workload, work pace, and multiple dimensions of quality of care directly address the stated aims and hypotheses. | Yes  Inclusion of a broad range of hospital employees across multiple departments within a regional hospital system is appropriate for examining workplace social capital and quality outcomes. | | Partly  The large sample size (n = 1589) and inclusion of diverse staff groups strengthen internal validity; however, the single‑hospital setting and moderate response rate (48%) limit generalisability to other healthcare systems. | | Yes  Validated instruments were used for measuring social capital, workload, and work pace, and quality of care was measured using structured self‑report scales informed by prior research, with acceptable internal consistency. | | Partly  A moderate response rate introduces some potential for non‑response bias, although dropout analyses suggested no major systematic differences. | | Yes  Binary logistic regression with adjustment for relevant covariates and marginal effect estimation was appropriate for examining associations between social capital subtypes and quality outcomes. |
| Lundholme et al (2022) Before–after evaluation  Overall judgement: The study meets MMAT criteria for quantitative non‑randomised research, with clear aims, appropriate intervention design, valid outcome measures, and suitable analyses; however, conclusions are limited by the single‑site setting, short follow‑up period, and reliance on self‑reported outcomes. | | Yes  The study clearly aims to evaluate whether participation in a medical escape room intervention improves workplace social capital among internal medicine intern residents. | | Yes  Pre‑ and post‑intervention survey data using a modified, validated workplace social capital scale directly address the study aim. | Yes  Inclusion of all first‑year internal medicine interns within a single residency program is appropriate for assessing an intervention targeting early trainee cohesion and social connectedness. | | Partly  The study included nearly all eligible interns (98% participation), strengthening internal validity; however, the single‑centre design limits generalisability to other residency programmes or specialties. | | Yes  The study used a modified version of a previously validated workplace social capital scale and supplemented quantitative findings with qualitative feedback, supporting construct relevance. | | Yes  Participation in the intervention was very high and completion of both pre‑ and post‑surveys was acceptable (80%), suggesting a low risk of non‑response bias. | | Yes  Paired t‑tests and chi‑square analyses were appropriate for detecting pre–post changes in workplace social capital scores following the intervention. |
| Mixed methods  Included studies | | Screening questions | | | Mixed methods studies | | | | | | | | |
|  | | Are there clear research questions/aims? | | Do the collected data allow to address the research questions/aims? | Is there an adequate rationale for using a mixed methods design to address the research question/aim? | | Are the different components of the study effectively integrated to answer the research question/aim? | | Are the outputs of the integration of qualitative and quantitative components adequately interpreted? | | Are divergences and inconsistencies between quantitative and qualitative results adequately addressed? | | Do the different components of the study adhere to the quality criteria of each tradition of the methods involved? |
| Havig et al (2013) Mixed‑methods study  Overall judgement: The study meets MMAT criteria for mixed‑methods research, with clear aims, strong methodological integration, and rigorous adherence to both qualitative and quantitative standards | | Yes  The study clearly aims to examine whether teams that meet an academic definition of “real teams” are associated with higher quality of care in Norwegian nursing homes. | | Yes  Data from staff surveys, manager interviews, relative interviews, and extensive field observations collectively allow assessment of both team functioning and quality of care. | Yes  A mixed‑methods approach is appropriate to distinguish between “real” and “quasi” teams and to capture multidimensional quality‑of‑care outcomes that cannot be measured using a single method. | | `Yes  Qualitative assessments (observations and interviews) are integrated with quantitative survey data through aggregation at ward level and combined in multilevel analyses. | | Yes  The authors clearly interpret how qualitative distinctions between real and quasi teams help explain quantitative differences in quality‑of‑care outcomes. | | Yes  Differences across data sources (staff, relatives, observations) are explicitly acknowledged and discussed as reflecting known proxy and perspective variations in nursing home quality assessment. | | Yes  Quantitative components use validated indices and appropriate multilevel analyses, while qualitative components are systematically collected through structured observations and interviews. |
| Killmeck (2021) Mixed‑methods observational study (Walk Rounds with structured observations and survey measures)  Overall judgement: Methodologically sound, with appropriate integration of observational and survey data, though limited by short implementation follow‑up and partial intervention fidelity. | | Yes  The study clearly aims to examine whether adding structured observations to Patient Safety Walk Rounds affects safety practices, safety climate, and teamwork climate. | | Yes  Direct observations, safety and teamwork climate surveys, and evaluation of action‑plan implementation collectively address the stated aims. | Yes  Combining in‑person observations, survey data, and implementation tracking is appropriate for examining both safety practices and climate outcomes. | | Yes  Observational findings informed action planning, and survey data were used to assess changes in safety and teamwork climate following implementation. | | Yes  The authors appropriately interpret the lack of change in safety and teamwork climate in relation to limited action‑item implementation and short follow‑up duration. | | Yes  The absence of climate change despite identified safety issues is explicitly discussed and linked to partial implementation and timing constraints. | | Yes  The observational protocol, validated survey instruments, and descriptive evaluation of action items are applied consistently and transparently. |
| Kile et al (2018) Mixed‑methods pilot study (education and cognitive rehearsal intervention)  Overall judgement: The study meets MMAT criteria for mixed‑methods research, with clear aims, appropriate design, effective integration, and transparent interpretation, though limited by pilot scale and short follow‑up. | | The study explicitly aims to evaluate the effect of education and cognitive rehearsal on nurses’ recognition of incivility, ability to confront it, perceived incivility, and job satisfaction. | | Repeated quantitative measures and qualitative open‑ended responses directly address recognition, response to incivility, perceived incidence, and job satisfaction. | A mixed‑methods approach is appropriate to assess both measurable changes in incivility over time and nurses’ subjective experiences of recognition, confrontation, and impact. | | Quantitative results and qualitative findings are presented together and used complementarily to evaluate intervention effects. | | The authors appropriately interpret quantitative reductions in perceived incivility alongside qualitative evidence of increased awareness and use of confrontation strategies. | | The absence of improvement in job satisfaction despite reductions in incivility is explicitly acknowledged and discussed as a limitation of the intervention and measurement approach. | | Quantitative components use validated instruments and appropriate longitudinal analysis, while qualitative data are analysed systematically using established content analysis procedures. |
| Literature reviews  Included studies | | Screening questions | | | AMSTAR 2 Literature reviews | | | | | | | | |
|  | Did the review include a clearly stated research question and inclusion criteria? | | Did the review authors use a comprehensive literature search strategy? | | | Did the review authors use a satisfactory technique for assessing risk of bias (RoB) in individual studies? | If meta‑analysis was performed, did the authors use appropriate methods for statistical combination of results and assess the potential impact of risk of bias on the results? | Did the authors account for risk of bias in individual studies when interpreting the results? | | Did the review authors provide a satisfactory explanation for, and discussion of, heterogeneity? | | If quantitative synthesis was performed, did the authors investigate publication bias? | |
| Velando-Soriano et al (2018)  The review demonstrates strength in clarity of research question, comprehensive searching, transparent inclusion criteria, and thoughtful discussion of heterogeneity. However, the absence of a formal risk‑of‑bias assessment limits confidence in the robustness of the conclusions, particularly given the reliance on cross‑sectional studies. | Yes  The review clearly stated its aim: to analyse the relationship between social support and burnout in nurses and identify risk factors. Explicit inclusion and exclusion criteria were defined, including population (nurses), outcomes (burnout measured using the MBI), exposure (social support), study type (primary empirical studies), and language restrictions. | | Yes  The authors searched five major databases (CINAHL, PsycINFO, ProQuest, PubMed, and Scopus), reported search terms, followed PRISMA guidelines, reviewed reference lists, and imposed no time or sample size limits to minimise publication bias | | | Partly  The authors did not conduct a formal risk‑of‑bias assessment using validated tools (e.g. Cochrane RoB or Newcastle–Ottawa Scale). Instead, they assessed study quality using Oxford Centre for Evidence‑Based Medicine (OCEBM) levels of evidence and degree of recommendation, which evaluates study design rather than internal bia | N/A | Partly  The discussion acknowledged limitations of the included studies, including predominantly cross‑sectional designs, self‑reported measures, heterogeneity in social support instruments, and methodological weaknesses. However, because no formal RoB assessment was performed, bias was not systematically integrated into interpretation of findings | | Yes  The authors extensively discussed heterogeneity across studies, citing variability in:  Social support measurement tools, Study designs (mostly cross‑sectional), Nursing settings and specialties, Cultural and organisational contexts  They clearly justified why quantitative synthesis was inappropriate and highlighted implications of heterogeneity for interpretation | | Yes  Because no meta‑analysis or quantitative synthesis was conducted, statistical assessment of publication bias (e.g. funnel plots) was not applicable. The authors instead attempted to reduce bias through broad database searching and inclusive eligibility criteria | |
| Campbell et al (2020) Literature review  Overall judgement: Methodologically sound integrative review with clear aims, comprehensive searching, and explicit appraisal of included studies. Limitations relate mainly to heterogeneity of study designs and outcomes and the absence of quantitative synthesis. | Yes  The review clearly states its aim and specifies inclusion criteria regarding population (RNs and NAs), setting (acute care), intervention focus (teamwork, delegation, communication), study type (intervention studies), language, and publication period. | | Yes  Multiple databases (CINAHL, MEDLINE, PubMed) were searched with clearly described terms, librarian input, reference list searching, and PRISMA‑guided reporting of the study selection process. | | | Yes  Methodological quality was assessed using established tools (MMAT for empirical studies; SQUIRE 2.0 for quality‑improvement studies), and appraisal results were reported. | Yes  No meta‑analysis was conducted due to heterogeneity in interventions, designs, and outcome measures | Yes  The authors acknowledge methodological variability and study limitations when discussing findings and drawing conclusions. | | Yes  Heterogeneity across interventions, outcomes, and study designs is explicitly described, with justification for narrative synthesis. | | Yes  Quantitative synthesis was not performed; potential publication bias was addressed indirectly through broad searching and inclusive eligibility criteria. | |
| Toles et al (2011) Literature review  Overall judgement: Methodologically sound narrative literature review with a clear focus and transparent search approach. Confidence in conclusions is limited by the predominantly descriptive nature of included studies and the absence of formal risk‑of‑bias assessment. | Yes  The review clearly aims to synthesise evidence on relationship‑oriented management practices in nursing homes and specifies inclusion criteria relating to setting, topic, language, and publication period (2000–2010). | | Yes  A structured search of PubMed using relevant MeSH terms is described, with screening of titles, abstracts, full texts, and hand‑searching of reference lists. | | | Partly  The review does not report a formal risk‑of‑bias assessment using a validated appraisal tool. Study quality is discussed narratively and indirectly through study design descriptions. | N/A  No meta‑analysis was undertaken; findings were synthesised narratively due to substantial heterogeneity in study designs and outcomes. | Yes  The authors acknowledge limitations related to predominantly descriptive designs, variability in measures, and limited intervention evidence, but do not systematically integrate risk‑of‑bias judgements into interpretation. | | Yes  Heterogeneity across study designs, outcomes, and management practices is clearly described and justifies the use of narrative synthesis. | | Yes  No quantitative synthesis was conducted; publication bias was not formally assessed. | |
| Moore et al (2019)  Overall judgement: Methodologically sound scoping review with a clearly defined purpose and comprehensive search strategy. Confidence in conclusions is moderated by the absence of formal quality or risk‑of‑bias appraisal, which is consistent with scoping review methodology. | Yes  The review clearly states its aim and research questions, focusing on the structures, processes, and outcomes of RN–PN collaboration in acute care hospitals. Inclusion criteria regarding population, setting, literature type, time frame, and language are explicitly reported. | | Yes  A broad, systematic search was undertaken across multiple databases, supplemented by hand‑searching key journals, professional nursing organization websites, and grey literature, with transparent reporting of the selection process. | | | No  No formal risk‑of‑bias or methodological quality assessment was performed, as per scoping review methodology. | N/A  No meta‑analysis was conducted; findings were synthesised narratively due to heterogeneity in study designs and outcomes. | Partly  While the authors discuss limitations related to descriptive designs, geographic concentration, and variable study quality, risk of bias is not systematically integrated into interpretation. | | Yes  Heterogeneity in study designs, outcome measures, and conceptualisations of collaboration is clearly described and justifies the use of narrative synthesis. | | Yes  Quantitative synthesis was not undertaken; publication bias was not formally assessed. | |
|  | | | | | | | | | | | | | |

| Papers that were not quality assessed | Type of paper |
| --- | --- |
| Kalisch et al (2005) Improving Nursing Unit Teamwork | Opinion |
| Hofmeyer et al (2008) Building social capital in healthcare. Conceptualizing collaboration in nursing organisations: Thinking ecologically for safer care | Opinion |
| Roth et al (2011) Essentials for Great Teams: Trust, Diversity, Communication ... and Joy | Opinion |
| Clark et al (2022) Civility: A concept analysis revisited | Theoretical discussion paper |
| Emich et al (2018) Conceptualizing collaboration in nursing | Theoretical discussion paper |

| Supplementary Table 3 show the contextual data that influenced our decision to assign the relationship behaviours to either ‘C’, ‘O’, or ‘M’. | |
| --- | --- |
|  | |
| **COM-B subcomponent** | **Supporting evidence i.e., direct extracts from each papers** |
| **Individual factors** | |
| **Reflective motivation** (beliefs about capabilities and consequences, role, identity, intentions, goals and optimism | *Positive attitude – showing respect*: The more positive way would be to say: I will **respect the judgments** of my fellow nurses, and I **expect them to give me that respect in return**. The more negative framing of this principle might be: **You leave me alone, and I’ll leave you alone**. The practice of mutual deference allows for offering help (Duddle and Boughton, 2007)  *Positive attitude* ***– showing respec****t:* Respect is imperative for collaboration. Collaboration requires working together with **open minds and valuing the contributions of each team member** (Schirm et al., 2000)  *Positive attitude:* Having a **positive attitude towards work and residents** and a **good companionship with colleagues** may make hassles [workload, staff levels] bearable and work more enjoyable. Care workers described that a positive attitude towards work **[i.e. being willing]** was important, because they were immensely influenced by each other and both positive and negative moods diffused through the entire workgroup. This supports Kelly and Barsade’s model of emotion in workgroups. Care workers both explicitly and **implicitly shared emotions through their daily interactions** and the groups affective states influenced the quality of their collaboration (Jakobsen et al., 2018)  *Positive attitude:* The average affective state of other team members over time affected the affective state of the individual team member. According to Walter and Bruch, workgroup emotion can be seen as an interdependent causal system where positive affective similarity and group relationship quality reciprocally combine to a dynamic, self-reinforcing upward spiral with both of these constructs strengthening each other over time. The care workers in this study actively sought to create this positive spiral, but were also aware that negative affective states could ruin their efforts. Research has shown that positive group-level emotion states led to improved collaboration, decreased conflict and increased task performance (Jakobsen et al., 2020)  *Positive attitude:* Maintaining a positive spirit of cooperation and mutual aid was highly valued by co-workers (Padgett, 2013)  *Positive attitude:* Training to increase staff “spirit at work” was related to improved communication and relationships.  (Toles and Anderson, 2011).  *Positive attitude:* The more mood regulation increases, the more the quality of health care improves (QOC). This suggests that a very pessimistic member, who does not look for maintaining a positive emotional atmosphere for himself and the team, can solely affect the whole group dynamic and lower its outcome. Emotional contagion of this member's “bad mood” could provide a possible explanation of this finding. Indeed, negative affect like stress spreads out particularly easily among nursing teams through a contagion phenomenon. The negative emotional state transmitted by this process could possibly alter team members' motivation to strictly respect safety and hygiene procedures, which in such an emotional atmosphere would be seen like tiring extra work. The higher the score of the “best” member of a team as regards Optimism/Mood Regulation is, the more the quality of health care increases in the whole unit. In line with the emotional contagion explanation mentioned above, one single member with a very high score on this dimension could, because of his or her optimism, frequent good mood, and concern for others' emotional well-being, improve his or her team's emotional atmosphere just by himself or herself. This team member would become a sort of emergent emotional leader, an “emotion manager” who pulls the team up. In such a positive ambiance, rigorously respecting safety and hygiene procedures could be perceived in a less constraining manner, and team members would therefore be more willing to make extra efforts. This hypothesis is akin to recent perspective on the role of emotion management on emergent leadership (Quoidbach and Hansenne, 2009). |
|  | *Values*: “If you don’t have the caring and willingness to show respect, you shouldn’t even get into the field because it’s something that has to be within you.” This response was echoed by many other nursing assistants. They felt that, usually, one cannot learn to provide good care unless one has an inner quality that makes being a nursing assistant much more than “just a job.” a good nursing assistant that included qualities of com passion, gentleness, patience, and personalised care. “You have to have patience, a lot of patience with some residents…and remember they are a person.” One nurse said, “Just knowing when someone needs a hug. Being caring leads to cooperation, cooperation among nursing assistants was identified as a factor that strongly affected quality of care. When the nursing assistants worked together and helped each other, the work - place was perceived as more pleasant and the quality of the work was perceived as better. "Little things that you learn, you just share them with each other. Mainly, we’re like a family. We all get along and we all help each other out (Schirm et al., 2000).  *Values*: Sharing personal values [with colleagues] may also lead to mutual understanding and service provision with guaranteed unity and continuity of care. Mu.tual understanding of the efficient progression of the job also develops quality (Norikoshi et al., 2018).  *Values:* Individual variations in personality, work styles, roles, and relationships were also described as affecting teamwork and team goals. NAP agreed, “it depends who came onboard on that shift whether you’re going to have a good day or a bad day.” (Bellury et al., 2016).  *Values:* Conscience can be regarded as central to a person and to play important roles within the person and the way they interact with others. Conscience can be perceived as an asset that helps care providers to provide good care (QOC) (Ericson-Lidman and Strandberg, 2015). |
|  | *Being willing:* The staff was asked to describe "good" vs "difficult" working relationships. Their stories provided rich details of practices occurring on patient care units. The qualities of good RN and UAP relationships included initiative, good communication, showing an appreciation for each other's contributions, and demonstrating a willingness to help each other with tasks. It is particularly significant to UAPs when RNs are willing to assist with direct patient care activities when the workload becomes busy (Potter and Grant, 2004).  *Being Willing*: At the same time, the effective functioning of the unit as a whole depended heavily on being willing to have extensive and informal cooperation. An intricate and careful dance was therefore required in order for this system to operate as smoothly as it did (Duddle and Boughton, 2007).  *Being willing:* There is a feeling that I want to help that person (staff member, patient or their families)’ (nurse manager, 30 years’ experience) (Norikoshi et al., 2018).  *Being willing:* The intervention (to improve connections with among co-workers) was associated with greater staff efforts to create a sense of community through being approachable, pitching in, seeking and accepting assistance, and engaging in teamwork (Colón-Emeric et al., 2014).  *Being willing*: Nurses willing to take part in collaboration must be willing to accept the group consensus (Schirm et al., 2000)  *Being willing:* Before collaboration begins, certain antecedents, or conditions, must be present. Initially, nurses need a willingness to participate in collaboration (Schirm et al., 2000).  *Being willing:* collaboration is a complex, relational process that involves more than nurses just working together on a team. While collaboration is a professional expectation, in practice, it nis a voluntary process among individual nurses. Simply, nurses can choose to collaborate or not and this barrier to collaboration has been identified in previous research. All it takes is one nurse with poor interpersonal skills and an unwillingness to collaborate and collaboration does not happen. The work of nurses is challenging because of working together for long periods of time, and with unpredictable and heavy workloads. Therefore, it important for nurses to demonstrate collegiality, a willingness to help each other when needed, and adaptability to change (Moore et al., 2019).  *Being willing:* The participants decided (after the intervention that empowered staff to engage in teamwork) that the factors guiding effective teamwork were goals worth striving for. After being provided with scientific knowledge about teams and teamwork, they concluded that the most important goal for both themselves and the residents was to develop their teamwork to increase the quality of care. All of the participants’ decisions on actions to be taken were guided by this conclusion. The results of this study showed that sharing and reflecting on knowledge within a team can start the process of improving team performance. In this study, troubled conscience related to deficient teamwork was used as a driving force for the improvement of care. The results of this study showed that sharing and reflecting on knowledge within a team can start the process of improving team performance (Ericson-Lidman and Strandberg, 2015). |
| **Automatic motivation** (emotions, reinforcements such as rewards, incentives and punishments) | *Psychological safety – having moral support:* “Moral support” was very commonly exchanged as well, and indeed this was part of what marked this unit (to most of the staff and to outsiders) as “one of the good ones”. Nurses commonly described their co-workers as their first and most important resource in daily work. While they did not describe this as a perfect system, they consistently reported generally high levels of cooperation and collaboration, and described this as one of the most important aspects of the work environment (Duddle and Boughton, 2007).  *Psychological safety – having moral support:* Nurses and CNAs also fostered collegiality through offers of moral support and assistance to co-workers they recognized as stressed by the challenging workload. One CNA described offering to help another in “one of the harder halls, where there are too many that you have to use the Hoyer that are total care that are totally the challenging workload. One CNA described offering to help another in “one of the harder halls, where there are too many that you have to use the Hoyer that are total care, that are totally (Madden et al., 2017).  *Psychological safety* *– having moral support:* Nurses intuitively perceived a sense of security with positive looks of others toward themselves: A new nurse said “I feel protected somehow at my workplace”’ (nurse manager, 30 years’ experience). Nurses were aware that they could use others’ strengths, information, knowledge and experience because nurses can overcome difficult situations: ‘Each one of the staff has a field of expertise. We should make good use of them’ (staff nurse, 15 years’ experience) (Norikoshi et al., 2018).  *Psychological safety - Trust:* Care workers explained that having a trusting relationship was connected with feeling secure enough to talk about problems at work or at home and that this in itself could alleviate stress symptoms. It also made it easier to ask for assistance if the work tasks became too much to handle (Jakobsen et al., 2020).  *Psychological safety - Trust:* Nurses mentioned the importance of trusting the staff’s potential abilities that will lead to better care. ‘It is important that one can feel secure about leaving it (work) to this staff member’ (staff nurse, 15 years’ experience) (Norikoshi et al., 2018).  *Psychological safety -Trust:* supported nurses’ and CNAs’ to show respect during their communication interactions. One CNA described trust as “about us personally because they are not always keeping an eye on us and watching us and it’s kind of cool because it makes us feel like they trust us enough to do our job well.” A nurse felt as though “most of my CNAs know me enough. I think the CNAs that I work with trust me. they know they can tell me.” (Duddle and Boughton, 2007).  *Psychological safety -Trust:* A strong, shared understanding of team work existed among NAP. Holistic patient caring was described as central to NAP teamwork and included listening and emotional support of one another led to trust and respect (Bellury et al., 2016).  *Psychological safety -Trust:* The interventions in this review focused on building a foundation of trust and respect through simulation, education and mindful communication. Examples of the interventions are team-based activities, such as TeamSTEPPS (Campbell et al., 2020).  *Psychological safety -Trust:* Trust in co-workers serves as an important psychological mechanism that allows employees to internalise the effects of a positive diversity mind set and helps to create a positive environment. Imagine a diverse nursing team in a large hospital. Expressing work relevant thoughts and feelings can be unexpectedly difficult because members usually have different opinions about a particular issue, and those thoughts stand a chance of conflicting with the views of others. Unless members trust in each other’s goodwill, it is natural for young and inexperienced nurses low in the hierarchy to take the path of “silencing”, which unfortunately sets members apart and threatens patient safety (QOC). These findings are consistent with the commonly shared wisdom; creating a trusting and “safe” environment is extremely important for high-reliability organizations, such as hospitals, which are characterized by task ambiguity, volatility, and high stakes, such as human lives and by salient power hierarchies (Lehmann-Willenbrock et al., 2012).  *Psychological safety -Trust:* We found that co-worker trust fully mediated the positive relationship between appreciation and nurse well-being, and partially mediated the positive relationship between appreciation and team commitment. Together, the findings provide suggestive, but not conclusive, evidence for the important roles of age diversity appreciation and co-worker trust in coping with age-diversity challenges and influencing nurse well-being and work attitudes (Lehmann-Willenbrock et al., 2012).  *Psychological safety –Trust*: An analysis of the authors' focus session interviews revealed that trust is central to effective RN and UAP relationships. Trust was a relational An analysis of the authors' focus session interviews revealed that trust is central to effective RN and UAP relationships. Trust was a relational characteristic formed from how the work of patientcare is organized and distributed among the RNs and UAPs (Potter and Grant, 2004). |
|  | *Positive reinforcement - affirmation:* Familiarity between staff, built by shared affirmation, helps engagement in duties with a positive feeling, facilitates various positive behaviours and ideas, and contributes to improving the quality of patient care. If colleagues in the workplace know each other well, trust between them increases (Kawachi et al., 2013) for the mutual benefit of all staff (Norikoshi et al., 2018).  *Positive reinforcement - affirmation:* Respect develops when RNs and UAPs share in the work of patient care and acknowledge each other's contributions (Potter and Grant, 2004)  *Positive reinforcement - appreciation:* As I’m in a campaign called ‘Thank you for calling’, I first say ‘thank you for talking to me’ when I speak with others’ (staff nurse, 10 years’ experience). ‘I always include appreciative words such as ‘thank you’ in conversation’ (staff nurse, 20 years’ experience) (Norikoshi et al., 2018). |
|  |  |
| **Psychological capability** (knowledge, decision-making processes, behavioural regulation) | *Resilience*: 'Highly resilient’ people understand the limitations of their capabilities at times of stress and are able to seek help when needed, which is an important part of the coping process. Resilient people tend to focus on getting past the difficulties and are able to apply skills gained in dealing with difficult situations to future situations. Resilient people have an increased ability to tolerate anxiety, frustration and distressing emotional experiences. Participants became increasingly resilient to the effects of interpersonal conflict in the workplace as a means of coping. Extremes of emotions, including screaming, yelling, swearing and crying, were an expected part of working life as nurse - it was distressing and that they spent varying amounts time thinking it over at home. Experienced nurses made a conscious decision not to take personally anything said during an outburst. In this way they were able to return to work and continue as if nothing had happened. Similar to the skills used in avoiding conflict, the ability to be resilient to emotional outbursts in the workplace was regarded by participants as something acquired over time. Some participants in our study had clearly been able to move on from the negative emotional experiences in their workplace. They had been able to adapt and persevere in the face of conflict, both around them and directed at them. This ability to be resilient or to bounce back helps individuals cope with adverse or changing circumstances.  *Resilience*: “Looking at negative feedback as an opportunity, instead of looking at it as being negative. It’s an opportunity.to improve on these things.” Other respondents believed nurses were not good at receiving feedback. For example, one respondent stated: I think it’s really important for nurses to be very much aware of their own attitudes, their own behavior. How they respond to feedback. A lot of us are very defensive when somebody gives us feedback and then pretty soon, people don’t want to talk to them, don’t want to give them feedback. And, then things happen that are not in the best interest of the patient or things happen that make it harder for everybody at work because everyone is tip toeing around that person. Each of us has to be aware of how we respond to negative feedback (Lux et al., 2014). |
|  | *Social competence –* Difficult workplace interactions between nurses appear to be part of everyday working life. Conflict is seen by managers and nurses alike as a normal part of the job and as such is usually tolerated. Experienced nurses navigated their way through conflict by developing skills to assess the potential success of an interaction before approaching another nurse. In many cases an angry confrontation could be avoided by careful surveillance of the environment i.e. observing colleagues from a distance, & deciding when the best time to approach. In paying close attention to the general atmosphere, the workload of others and what was happening for each individual, nurses could protect themselves from potential conflict by not inflaming the situation further. Absorbing the atmosphere, allowed RNs adjust their behaviour to suit the situation. Experienced nurses also recognized that this was a process they had learned over time, to a point where it became something that they did not think about; it became normal behaviour and less experienced nurses had often not developed these skills. Inability to read the signs of an impending conflict increased the likelihood of being subjected to an angry outburst from another nurse. It was often perceived as very difficult to speak to RNs about their practice without it been taken in the wrong way. When they did offer feedback, the nurses said they were very careful how they framed it; they described their efforts in ways that emphasized their attentiveness to these social conventions and relationships. One fairly new nurse, for example, was especially sensitive to the way her feedback might be taken by more senior staff. Many of the nurses used very similar language, emphasizing that feedback, if given, would need to be said in the right way – “not harsh”, “not in an accusing way.” However, the simplest and perhaps most common strategy described was to not say anything at all. “I just swallow a lot,” said one nurse. “I just let it go – it’s not worth it,” (Duddle and Boughton, 2007).  Social competence - involves the ability to evaluate social situations and determine what is expected or required; to recognize the feelings and intentions of others; and to select social behaviours that are most appropriate for that given context. Because of the respectful nature of their relationship, if an occasion arises where the GN knows about the most recent research evidence regarding a particular condition, they are tactful in the way they impart their knowledge to the EPN. The above illustrates the tactfulness of the GN in reassuring the EPN about her use of evidence-based practice. The GN was able to translate what evidence-based practice meant to the EPN using an example that the EPN would be familiar with. There was no evidence of power (Hoare et al., 2013).  *Social competence –* Results showed that health care quality was positively correlated with emotion regulation. Emotional Intelligence (EI) and, more particularly, the ability to deal with one's own emotions, allowed team members to be more inclined to listen to alternative viewpoints and to look for superior solutions, without feeling threatened by the possibility of being wrong. the same authors reported that the mean level of EI in a team affected its conflict resolution style: Teams with a high level of EI had a preference for collaborative conflict resolution strategies, whereas teams with low EI preferred avoiding strategies (Jordan & Troth, 2002). EI seems to be a facilitating factor for group cohesion. In this way, according to team members who are emotionally intelligent form strong relationships and a solid team support system. Moreover, empathy that shares close relationships with EI also has been argued to be an important characteristic necessary for team cohesion. The lower the EI score, “the worst” team member regarding Optimism/Mood Regulation is, the lower the quality of health care provided by the whole team is. Emotional Regulation may provide an interesting new way of enhancing patient/client outcomes and training future professionals to this ability could be especially fruitful. EI could constitute an interesting new way of building cohesive teams in organizations. Including training on emotion regulation skills during teambuilding seminars might be more effective than bungee jumping to create long-term cohesiveness. Concerning relationships between emotional Intelligence (EI) and cohesiveness, high-EI groups are significantly more cohesive than low emotional Intelligence groups. Emotional Intelligence thus seems repeatedly to play a part in team cohesiveness. Team members' ability to seek for or to maintain positive emotional states in self and others helps them in getting along and pursuing common goals (Quoidbach and Hansenne, 2009)  *Social competence – awareness:* The intervention improved the teams ability to give feedback and deal with conflict. RN: We are much more likely to tell each other when there is a problem. We used to pass it to the manager (Kalisch et al., 2007).  *Social competence – awareness*: Our results underscore the need for individual staff members to be attentive to their troubled conscience. To constructively deal with their troubled conscience, the participants expressed and  interpreted its message with each other and took actions that they perceived to be in the right direction (Ericson-Lidman and Strandberg, 2015).  *Social competence – anticipate and adapt*: Nurses perceived whether looks from others were positive or negative and used this perception to closely measure the workplace environment. This is similar to the concept of cohesion, with a related workplace social capital attribute being affirmation (Jung et al., 2011, 2012)....Nurses were aware that through the conscious and skillful use of staff members’ strengths (information, knowledge and experience), high quality nursing may be provided and difficult situations overcome. Nurses also considered that they should consciously perceive themselves as role models, and they must be aware of their roles to promote others’ growth. This is similar to an individual’s ability to access valuable social positions within a network (Lin, 2002) and the nurses perceived the significance of conscious use of ‘access to the strength possessed by others’ that was manifested....Because imbalances within work roles produce discontent, nurses tend to create win–win relationships with others. This emerged through nurses acting while considering the benefit to others first, with special attention directed to being fair. In addition, nurses displayed conscious consideration of others’ roles outside the organisation (e.g., motherhood). This altruistic reciprocity may also be considered as analogous reciprocity (Kouvonen et al., 2006), which is another attribute of WSC, with the exception of prioritizing others’ needs (Norikoshi et al., 2018).  *Social competence:* RNs quickly had to adapt into the culture of their new ward & began to learn the unspoken rules including which specific clinical duties should be performed, unspoken rules of behavior (i.e. what was and was not acceptable). Understanding/adapting to the atmosphere of an assigned ward was the most difficult aspect of transitioning from nursing student to new nurse. New nurses were required to abide by these rules regardless of their personal thoughts. Objections to or behaviours deviating from these rules were not tolerated, and there was a tendency to label new nurses as strange if they breached these boundaries (Kim and Oh, 2016)  *Social competence – anticipate and adapt:* Individual variation was mentioned within well-defined groups of RNs, such as new graduates or preceptors, novice or seasoned nurses, and in the extent to which RNs were flexible, helpful, and adaptable in daily work. These differences were essentially attributed to individuals rather than RNs as a group (Bellury et al., 2016).  *Social competence – anticipate and adapt:* Nurses need to possess effective communication skills, interpersonal skills to adapt to difficult situations to work as a team. The importance of a common vocabulary is noted (Schirm et al., 2000)  *Social competence*- *techniques*. Make a conscious effort to stop, watch, and act. With the CONNECT they are saying, now listen, we got to do some two-eyed seeing [CONNECT communication technique] here, our strategies got to be different, we’ve got to be able to listen, as well as be approachable, as well as ask the right questions to get the information that you need to take care of the resident. Because it is not about us. So, I think more than anything that the CONNECT put that in focus for us because we were having some difficulties at that time with some of our team members (Colón-Emeric et al., 2014).  *Social competence*- *techniques*. The collaboration was influenced by the interpersonal skills of the nurses and included the sub‐themes of communication skills, shared decision‐making, and relational skills. Interpersonal skills are verbal and non‐verbal communication, active listening skills, relational skills, cooperation, and decision-making skills. Most of the studies showed that the process related theme and interpersonal skills could positively or negatively influence collaboration. The interpersonal skills most frequently associated with positive RN‐PN collaboration were communication skills. The verbal communication among the nurses related to patient care decisions. The relational skills of the nurses was an important sub‐theme. Relational skills are the ways in which individuals or groups interact and connect. Developing professional relationships or what was referred to as ‘working harmony’ of RNs and PNs was highlighted as an important contributor of collaboration by several authors. The nurses demonstrated positive relational skills by being cooperative, willing to help other nurses when needed (eg. providing “backup”), being open‐minded, flexible, and adaptable to change, having conflict management skills, and having mutual trust. Alternatively, the poor relational skills of RNs and PNs negatively impacted collaboration. Examples included: negative attitudes about current or prior conflict and resistance to try new models of team nursing care, and uncooperativeness relating to RN supervision or delegation of PNs (Moore et al., 2019)  *Social competence – techniques:* Being able to transfer of information along with authority and responsibility during transitions in care across the continuum; to include an opportunity to ask questions, clarify and confirm (Thomas and Galla, 2013)  *Social competence - cognitive rehearsal training* - was an intervention that teaches staff what incivility is and how to respond. The statements (i.e., “try to monitor body language,” “minimize/halt discussions of co‐workers,” and “self‐awareness of my own actions”) indicate the participants recognized and confronted uncivil behavior (i.e. were socially competent after training). This study adds to the body of knowledge and supports previous study findings regarding the effectiveness of education and cognitive behavioural techniques in increasing nurses’ awareness and ability to confront of uncivil behaviours. Repeated training and education is recommended for sustainability. Healthcare organizations are encouraged to invest time and dollars in civility training for managers and nursing staff (Kile et al., 2019) |
|  |  |
|  |  |
| **Organisational** | |
| **Social opportunity** (societal influences such as social pressure, norms and comparisons) | *Culture – team norms:* Perception of the organizational environment takes on personal meaning and significance on employees. When employees perceive their work team positively, they are likely to identify their personal goals with those of the organisation and to put greater effort into pursuing them. Employees who identify with their proximal work group and interact with colleagues are likely to develop shared patterns of understanding and norms of behaviour which allows a shared climate to evolve (Heponiemi et al., 2012).  *Culture – team norms:* When performed well, nurses and CNAs described the result in symbolic terms such as a “beautiful routine,” “like dancing,” or “a smooth-running machine.” (Madden et al., 2017).  *Culture – team norms:* group norms include communication patterns, values and beliefs, and traditions Assuring that nursing students do understand the culture of the workplace and preparing them to address cultural practices that are negative are an essential role for co-worker relationships (Lux et al., 2014).  *Culture -team norms:* Some procedures were expectations defined by institutional policies; others were guided by uncodified norms (cooperation and taking initiative) that arose through an understanding of shared values.....“No matter what job you do, you need to perform and follow the rules.” A nurse indicated, “You have to have expectations with your staff and your CNAs . and if you don’t communicate with them clearly, things don’t get done.”....Policy-based and norm-based communication processes were intricately connected; poor performance in one area negatively influenced performance in the other. When performed well, nurses and CNAs described the result in symbolic terms such as a “beautiful routine,” “like dancing,” or “a smooth running machine.”.....A nurse described her role as “their [CNAs] supervisor and they have to report to me.” A CNA explained, “They [the nurse] can tell us what we need to do and we’re supposed to listen to them.” One CNA explained, “No matter what job you do, you need to perform and follow the rules.” A nurse indicated, “You have to have expectations with your staff and your CNAs . and if you don’t communicate with them clearly, things don’t get done.” (Madden et al., 2017).  *Culture: team norms:* the intervention created a norm of open communication: “A lot of times CNAs have their own section and it seemed like…not that they were afraid to ask for help, but they didn’t want to bother somebody else that was busy doing something. But after that role playing, everybody . . . said what I’ve always kind of felt like, I couldn’t ask somebody for help. But now I feel like I can, is normal, because we are all kind of feeling that same thing, you know . . . They just felt more comfortable about asking for that help if they need it (Colón-Emeric et al., 2014).  *Culture: team norms*: Mutual respect for each healthcare team member’s role and profession is necessary for effective collaboration. Team members must value what other members of the healthcare team bring to the discussion including the roles and knowledge of the team members. Respect requires healthcare teams to forego any hierarchical structure and allows all members to be equals (Schirm et al., 2000).  *Culture – team norms:* The intervention created greater team awareness of one another and more back-up behaviours (RN: We are working really well with each other. It is a mentality of "our patients" not just "my patients"; RN: We are more aware of each of their problems and issues, more open to helping each other than before; CNA: People are finding out more about each other. RN: The CNA/RN relationship is better; CNA: Nurses are helping out more with patient care. Nurses will help with things i cannot do on my own, like turning a patient or bathing a patient. The appointment of a guiding team made up of unit staff and managers who engaged in creative idea generation, testing, and implementation of ideas for change; a comprehensive communication strategy to keep the entire unit staff informed and involved in the project; and follow up after training by managers and guiding team members to reinforce the new behaviours and ultimately change the culture of the unit to one that supports teamwork (Kalisch et al., 2007).  *Culture – team norms:* How group members held each other accountable for the content and quality of their  verbal and nonverbal communication became the intended focus of long-term work for every group (DiMeglio et al., 2005)  *Culture – team norms*: advocate for the patient; this is invoked when team members’ viewpoints do not coincide with that of the decision maker. Assert a corrective action in a firm and respectful manner; make an opening, state the concern, offer a solution and obtain an agreement.  *Culture – social support:* The importance of a positive workplace atmosphere, having fun together and trusting each other. [Social relations with colleagues] is what matters most. Of course, we need to work and of course we need to educate ourselves and become better professionally, but it just does not work if we cannot talk to each other and have a little fun sometimes. That is essential, preventing larger conflicts to arise. Having a positive relationship intertwined with care workers descriptions of how a positive atmosphere eased stressful situations, prevented conflicts with residents and made work more enjoyable. Care workers accepted that their work at times could be busy and possibly stressful due to the nature of work tasks. Working alongside colleagues who took this as a positive challenge and with confidence that they would still be able to provide relevant and adequate care made it easier to keep a positive attitude themselves. I think that the first 15 minutes of the shift, how the atmosphere and mood is, when you come to work [is important]. If there’s a calm and positive atmosphere, it doesn’t really matter if the whole house topples over, but if you enter the door and get this negative energy sent at you all the time, then it affects the rest of your shift. And I think that it’s sometimes culture borne. Things become a habit (SH, Tulip care centre). A positive atmosphere should, however, not merely be seen as something relying on the social relations among care workers, but also something that care workers should work on as part of their professionalism by focusing on problem-solving rather than blaming each other when problems occur (Jakobsen et al., 2018).  *Culture – social support*: It is generally agreed that the most important support is that provided by the supervisors and co-workers. When there is little or no exchange of ideas and experiences with co-workers, when relationships with supervisors are poor and when there is a lack of positive feedback, then burnout can more readily emerge. Social support in positive environments minimizes feelings of isolation that can arise when problems arise in nurses' day-to-day work and helps create a network for the exchange of experiences and the learning of coping strategies to deal with stress inducing situations. A network of SS has a positive effect on nurses' working lives, by giving them a sense of security and enhanced self-esteem. Nicholson et al. (2014) conducted a longitudinal study of the DP-civility and DP-confidence relationships. These authors concluded that although the level of confidence expressed by co-workers had no impact on DP, the absence of civility from co-workers was predictive of high levels of DP, approximately 1 year later (Velando‐Soriano et al., 2020).  *Culture – social support*: the creation of social support-oriented groups can act as a buffer against work related stressors. Such groups could discuss problems arising in the workplace and help resolve them or reduce their impact. SS from co-workers is a prevent emotional exhaustion and depersonalisation. Nurse supervisors who had few opportunities to regularly meet their fellow nurses presented higher levels of Emotional exhaustion (Velando‐Soriano et al., 2020).  *Culture – social support:* As shown in other studies, it is vital to have a supportive and positive work culture, which promotes intra-professional collaborations (Wei et al., 2020). This review confirms that interventions to improve teamwork, delegation and communication can influence the relational qualities between RNs and NAs. For RNs and NAs to collaborate and form a highly functional team in hospital settings (Campbell et al., 2020)  *Culture –social support:* Social Support from co-workers is a coping strategy to prevent Emotional Exhaustion and Depersonalisation. Such coping strategies should be included in training programs for nurse supervisors (Velando‐Soriano et al., 2020).  *Culture – social support:* Feelings of being in a safe and well-functioning environment were closely linked with helping each other with practical work tasks. This is akin to a study by Semmer et al. who have shown that offering practical help communicates care and acceptance and thereby supports the sense of belonging. In this sense, social relations can be seen as a basis for professional collaboration. Strong social bonds with colleagues also allow care workers to receive emotional support when facing challenging residents, a heavy workload, or private problems. In addition, organisational psychology has shown that high quality relationships are a source of well-being for people at work (Jakobsen et al., 2018).  *Culture – social support*: Participants’ troubled conscience was eased through reflecting on and sharing their thoughts about their teamwork. They emphasised that they had, with support, started their learning process into what teamwork was about. They had identified the shortcomings of their teamwork and also raised their awareness of their abilities and resources to deal with problems in a fruitful manner, in this case how to gradually improve their teamwork (Ericson-Lidman and Strandberg, 2015).  *Culture –safe environments:* Nurse managers must create safe environments to build and maintain dynamic teams of RNs and NAs. Communication, delegation and teamwork take time and focused attention and require nurse managers to intentionally foster relationships of trust and respect between the RN and NA (Campbell et al., 2020)  *Culture –safe environments*: Trust builds when UAPs know they are valued as part of the nursing team (Potter and Grant, 2004)  *Culture - feeling heard:* Nurses and CNAs perceived that receiving a response was a basic indication that they were regarded by their coworker. One CAN explained, “At least I know that I have been heard, when they [coworkers] say, ‘Okay, thanks for letting me know,’ or ‘I heard you, I got that.’” In addition to simply being heard, nurses and CNA appreciated when the information was heeded. Phrases, such as “I’ll take care of it,” “I’ll write that down,” or “OK, I’ll go check it out,” were given as examples of coworker responses that indicated that what they said mattered (Madden et al., 2017).  *Culture - feeling heard:* When RNs’ communication was not directly acknowledged, they assumed their communications had been ignored. It was therefore important to feel heart. RNs’ statements, “we are in this together” and “we make it work.” Additionally, RNs seemed to expect frequent communication. One noted, “Throughout the shift we will report to each other as needed.” Although both RNs and NAP acknowledged the importance of information exchange, the busyness of a complex work environment regularly precluded closed-loop communication in favour of one-way communication, or telling (Bellury et al., 2016)  *Culture – feeling heard:* Staff in focus groups talked about the importance of making all staff feel involved and valued so that they would come to management with information. Control Licensed Practical Nurse (LPN): Now that I’m part of this team my opinion counts. I’m being educated on what I need to do and with this falls committee we can take it another step further. We can take it to restorative . . . we can take it to incontinent care. It’s a different level.... Intervention Housekeeper: In the beginning. . . . it was like, “why is she making us go to these meetings?” ‘Cause I’m figuring, I mean seriously, I’m a housekeeper, what do you need me for? And I’m laundry, so why? But after we got into [the program], it was like, “Oh, I am a piece of this puzzle. We are!” Because I really didn’t think that we were (Colón-Emeric et al., 2014). |
| **Psychological capability** (knowledge, decision-making processes, behavioural regulation) | *Mentorship - increasing knowledge, awareness and confidence:* Collegiality was fostered most often through communication associated with mentoring one another job during resident care. A CNA explained that, “For a lot of CNAs, this is their first job, that is why they don’t know, they are learning.” Nurses mentored CNAs who “through no fault of their own” were poorly trained with limited experience. Sometimes they [CNAs] don’t understand how to do something or what they should do. I usually step in and give them some more explanation, some teaching if they don’t seem to understand what I am asking them to do, or I say OK, come and I will show you what to do. Experienced nurses mentored other nurses with less experience in resident care and work processes (Madden et al., 2017).  *Mentorship - increasing knowledge, awareness and confidence:* Using mentors during this transition from University to practice was one means of assisting students to understand the culture of the workplace. Mentors have helped me deal with difficult relationships by being objective in their outlook and helping me see the relationship in an objective way. Understanding the culture of the workplace was described as an important aspect of transition to professional practice. Nurse educators must take the lead in educating nursing students about how to confront DB before they enter practice (Lux et al., 2014).  *Mentorship - increasing knowledge, awareness and confidence:* Certain aspects of the job cannot be learned through any means other than on-the-job experience (Schirm et al., 2000).  *Mentorship - increasing knowledge, awareness and confidence:* Other researchers, too, have suggested that appropriate support from supervisors can limit the development of burnout, and that programs should be implemented to help nurses manage stress, develop coping abilities so they can increase knowledge and skills of their subordinates (Velando‐Soriano et al., 2020).  *Mentorship - increasing knowledge, awareness and confidence:* Relationship-oriented management practices are not the exclusive purview of managers. They can be fostered as a grassroots effort, begun by clinical staff without direction from managers. This suggests that staff nurse education should include learning how to foster effective interdependence and confidence (Toles and Anderson, 2011).  *Mentorship - increasing knowledge, awareness and confidence:* Good teamwork must be encouraged by supervisors in order to increase empowerment among team members and teams work when all who have influence over a patient or resident’s care are included on the team. Belief that one’s supervisor includes and mentors them as a member of the health care team was a significant and positive predictor of Total Empowerment and all dimensions except Responsibility. Future work might focus on developing targeted tools and trainings to assist specified team members (supervisors, etc.) in their ability to include or engage subordinates or co-workers in caring for residents (Barry et al., 2019).  *Mentorship - increasing knowledge, awareness and confidence:* Opinion leaders such as nurse practitioners and  trusted colleagues can play a vital role in fostering the flow of quality information across the bonding, bridging and linking social capital relationships in the health care organization and beyond (Hofmeyer and Marck, 2008).  *Leadership: holding staff accountable*: Collaboration among health care providers is considered a strategy to provide optimal patient care (QOC). Nurse administrators and/or managers play a significant role in developing, promoting, and evaluating collaborative relationships among nurses. Nurse managers must also be willing to hold individuals nurses accountable if they are not demonstrating the necessary attitudes and behaviours required for successful collaborative practice. Organizations should consider using a model or framework to guide nursing collaborative practice (Moore et al., 2019).  *Leadership: holding staff accountable:* When staff disregard organisational value statements about respecting difference and diversity, nurse leaders need to reward those who speak up about forms of retribution, intimidation and bullying rather than remaining silent, thereby encouraging more and more colleagues to act as a collective moral compass for the organisation. Setting this standard of organizational integrity and personal responsibility also positively influences the clinical educational experience of all students within the organization and informs their leadership development for the future. Nurse leaders can also support teams to speak up about moral matters in group conversations by sharing stories from practice within the safety of an Ethics in Practice session, incident debriefing or other safety-oriented activity (Hofmeyer and Marck, 2008). |
| **Physical capability** (skills/abilities or proficiencies acquired through practice) | *Leadership – conflict resolution skills:* Managers were key in making care worker collaboration work. Managers were not themselves a part of the day-today collaboration, but they provided the managerial framework for the day-to-day work tasks of the eldercare workers and provided trouble-shooting interventions. Managers could facilitate empowerment by supporting care workers in solving the problems and coordinating directly with one another. According to the care workers, conflict resolution was an important part of the manager’s job description, but not all managers possessed the necessary skills. If managers were not able to make a clear decision and deal with conflicts in the group, it had negative consequences for collaboration within and between workgroups. These were skills that needed to be learned in line with other management skills. The focus group discussions revealed that skills varied greatly across managers within the same eldercare home (Jakobsen et al., 2020).  *Leadership – conflict resolution skills*: The manager instigated a two-challenge rule - A constructive approach for managing and resolving informational conflict; when we have different information. When an initial assertion is ignored; it is your responsibility to assertively voice your concern at least two times to assure it has been heard. The team member being addressed must acknowledge. If the outcome is still not acceptable take a stronger course of action and/or utilise a supervisor or chain of command. Empowers all team members to ‘stop the line’ if they sense or discover an essential safety breach (Thomas and Galla, 2013).  *Leadership – conflict resolution skills*: Nurse leaders are responsible for creating a positive work environment for staff, providing safe care to patients, and supporting fiscal goals of their organizations. Nurse‐to‐nurse incivility can negatively affect each of these areas. This pilot study adds support to the evidence that education, and the use of cognitive behavioural techniques is an effective method for increasing nurse leaders awareness and their ability to confront uncivil behaviour leading to a decrease in the incidence of this behaviour. Teaching nurse leaders about incivility and providing them with tools to confront this behaviour is necessary (Kile et al., 2019).  *Leadership – shared mental models:* Without a shared language for professional group practice (denoting shared decision-making and responsibility), the staff on the unit identified themselves simply as people who helped each other and got along well (Padgett, 2013).  *Leadership: shared mental models:* shared mental models (thought processes) have been conceptually defined as “individually held knowledge structures that help team members function collaboratively in their environments” proposed that teams need shared mental models related to team goals, team member tasks, and coordination of the team (Bellury et al., 2016).  *Leadership: shared mental models:* Each group reported a lack of respect by the other. Other researchers also have supported the need for shared values for creating high-quality working relationships, trust, and reciprocity. The NAP-perceived lack of respect seemed related in part to differences in education, as seen by DiBenigno and Kellogg (2014), who concluded demographic differences between work groups led to status differences and conflict in health care delivery (Bellury et al., 2016).  *Leadership: shared mental models:* Nurse leaders conveying a supportive culture that promotes teamwork was a positive influencing factor. Having shared “mental models” or mutual professional beliefs, values, or philosophies for patient care had a positive influence on RN‐PN collaboration (Moore et al., 2019).  *Leadership: shared mental models:* The intervention involved the entire staff team to ensure shared mental models, values, vision and goals of the project to improve teamwork (Kalisch et al., 2007).  *Leadership: shared mental models*: Three conditions need to exist for sharedness to be possible: individuals must interact in the work, there needs to exist a common goal or attainable outcome that predisposes individuals towards collective action, and there needs to be sufficient task interdependence to develop a shared understanding (Heponiemi et al., 2012).  *Leadership: shared mental models*: While age diversity [among staff] can lead to increased creativity and a greater richness of values and skills, it can also lead to value clashes, disrespect of each other’s viewpoints, and increased conflict. We propose the injection of positive diversity shared mindsets (age diversity appreciation) as one remedy. Specifically, we suggest that age diversity appreciation is positively related to nurses’ well-being (stress and work–life balance), and also positively related to their team commitment (Lehmann-Willenbrock et al., 2012).    *Leadership: shared mental models*: The research on teams illustrates that high performing teams have a common purpose and a common destiny. Although nursing staff generally believe they have a common purpose which is to provide quality care, they do not see their purpose as providing quality care 24 hours a day and to all of the patients on the unit. Instead, they assume accountability for only their patients on their shift. Many nurses do not even assume responsibility for the work of the assistive staff who work under them. If the nursing staff is only focused on taking care of their patients on their shift, a high-performing team cannot be achieved. Added to this problem is that nursing staff do not have what is called a common destiny. Common destiny means that all members of a team share equally in their successes and their failures. If a patient is dissatisfied with their nursing care, who is held accountable? (Kalisch and Begeny, 2005).  *Leadership:* *being tactful*: When they did offer feedback, the nurses said they were very careful how they framed it; they described their efforts in ways that emphasized their attentiveness to these social conventions and relationships. I think everyone has their own style of nursing, and I’ve learned that there’s really no right or wrong way (Padgett, 2013).  *Leadership: role modelling behaviours:* One nurse noted that supervision of nursing assistants included imparting knowledge about team work, saying, “I always tell my assistants….we need to work as a team. The better we work as a team….the better everybody feels about it [and the residents] get the best care they need” (Schirm et al., 2000).  *Leadership: role modelling behaviours:* Nurses also mentioned the importance of taking responsibility for supporting others’ careers when making the most of their field of expertise: ‘Each one of the staff has various drawers (fields of expertise), so I want him or her to act as a role model for the others’ (nurse manager, 25 years’ experience).‘I try to tell staff nurses about my values, nursing views, and ethical views’ (nurse manager, 20 years’ experience)....‘Although I’m only a staff member at work, I play a mother’s role after I go home, therefore I need to consider everything when I speak with a person’ (nurse manager, 30 years’ experience)....‘I feel that the negotiation skills of our department chief are a win–win but altruistic’ (director of nursing, 35 years’ experience)..(Norikoshi et al., 2018).  *Leadership: role modelling behaviours:* Results from this review support the importance for nurse leaders to find ways to build partnership and respect between RNs and NAs. Nurse leaders are anchors of a work unit and play a significant role in modelling behaviours that promote a healthy work environment and interpersonal relationships (Campbell et al., 2020)  *Leadership: role modelling behaviours*: Nurse managers are essential in evaluating care delivery provided by RNs and NAs to identify gaps in relational quality and determine the potential influence on patient outcomes. Leaders should consider their role in bridging these gaps. Observing bedside shift reports, participating in walking rounds and listening to the concerns of the RN-NA team may be a way to build rapport and decrease silos (Campbell et al., 2020).  *Leadership: role modelling behaviours:* Management cultivation of staff relationships was related to better uptake of evidence based practice guidelines (QOC). Management that was open, respectful, and helpful was related to greater nursing assistant intent to stay; (QOW) nursing assistant intent to stay was also related to resident satisfaction with care (QOC). relationship oriented management fosters staff interdependence, supported by stronger social networks and more open communication channels among nursing-home staff and managers. Establishing effective interdependence is, in large part, the work of managers in nursing homes, suggesting a need to include relationship-oriented management in nursing administration education programs (Toles and Anderson, 2011).  *Leadership: role modelling behaviours*: Leadership is a key factor in improving team effectiveness, that is, to uphold quality of care (QOC). The first-line manager role in the care of older people is central to both healthcare and social care quality and for employees’ work environment. However, the leadership role is also problematic as they are squeezed between managers on a higher level and politicians, who are expecting them to manage the organisation, on the one hand, and employees and care recipients, who expect them to lead, on the other hand. Recognising the importance of a present, supportive, interested, and motivated manager to achieve good teamwork. Staff wanted the manager to be present at the facility-unit meetings in order to jointly discuss their teamwork and to enable their participation in decisions regarding residents’ needs and other issues. The manager agreed to be present and thought it was a constructive idea worth testing. We would like our manager to be present on some of our meetings, we have a need to communicate in a deeper sense with her, for example about the residents’ different needs...we hardly ever see her. Seems like a good idea, if you want me to be present I’m ready to test this meeting form. Through the intervention had increased their awareness that the team was to be regarded in a broader sense and that they were in need of a present manager and RN for their support, competence and mandate. A need to develop a closer cooperation between the ENs, NAs and RNs was expressed) (Ericson-Lidman and Strandberg, 2015).  *Leadership: role modelling behaviours*: This paper examined the quality of supervisor–subordinate relationships teamwork, wellbeing, affective commitment, and turnover intention for nurses. Commitment to the role, turnover intentions are influenced by the quality of supervisor-subordinate relationships, teamwork and wellbeing. The study used a Social Exchange Theory framework that argued that the ideal situation is one in which all nurses are satisfied with their supervisor– subordinate and colleague–colleague relationships because this would mean that they are sharing their time and other resources such as information, knowledge and skills, support and assistance with one another. Under such conditions, nurses would assist one another during busy periods, thereby reducing stress and burden, and it is likely that this would have a positive impact on their perception of wellbeing and commitment to the hospital. These findings suggest that management must focus on improving the quality of workplace relationships as a first step in retaining skilled nurses (Brunetto et al., 2013)  *Leadership: promoting role clarity:* An equally important antecedent of collaboration is knowledge of one’s roles and skills as well as the roles and skills of others. Knowing the roles and skills of each member of the healthcare team allows for contacting the person best equipped to aid in solving the identified problem (Schirm et al., 2000).  *Leadership: promoting role clarity:* Role clarity is an important component of effective collaboration. If RNs and NAs are unsure what to do and struggle in silos, they will be unable to bridge the gaps in patient care. Nurse managers must facilitate effective delegation practices between RNs and NAs while maintaining relational quality between the RN and NA (Campbell et al., 2020).  *Leadership: promoting role clarity:* Collaboration is stronger when nurses understand not only their own roles but also each other’s roles. Organizational support and policies and having clear role descriptions, accountabilities, and responsibilities were considered an important contributor to collaboration. The subsequent two sub‐themes revealed the factors that negatively influenced RN and PN collaboration including role ambiguity and unsupportive leadership. Role ambiguity was a barrier to collaboration as it created confusion. The review identified that RN‐PN collaboration was facilitated when the SOP of nurses was clearly delineated. Hospitals using a collaborative/teamwork model or framework assisted with RNs and PNs understanding their scope or practice and positively contributing bto their collaborative practice (Moore et al., 2019).  *Leadership: promoting role clarity:* The intervention led to role clarity. Clarification of roles of team members (RN: I feel like everyone is much clearer about our roles. The is much less of this feeling that CNAs think we goof off when we are doing paperwork (Kalisch et al., 2007). |
| **Physical opportunity** (environmental context) | *Composition of teams*: In the nursing home unit, the head nurse had introduced a number of measures to ensure openness and collaboration through what we may call ‘an organized togetherness’. An example was how the head nurse had linked the auxiliary nurses in pairs to be each other's primary discussant when care plans were to be made and revised. One's ‘partner in the pair’ would also step in as the substitute primary contact for the other's two or three residents if the other were on sick leave or on holiday. The auxiliary nurses said that it was very fruitful and reassuring to have a permanent partner with whom to discuss various issues. When it is like that, it is more intimate or a little more like belonging. It’s a little more like – then they know. It’s not vague. Then they know that they’re a team and that they have to take care of each other’s duties and each other’s patients. Like, if someone is on holiday and she forgot to do certain tasks before she left, then the other (her partner in the pair) has to take care of those things. (…) That may create some tensions, and the other may think, ‘Why did you not do that (yourself)?’ Like, you know what I mean? (Munkejord, 2019)  *Composition of teams*: How RNs and UAPs are assigned to work together has direct bearing on the type of working relationship they develop. When a UAP is assigned to work for multiple RNs during a given shift, the RNs and UAP do not partner or work together in ways that are necessary to build trust and familiarity with one another's work habits. In contrast, one-to-one RN and UAP assignments promote working conditions that foster the development (Potter and Grant, 2004).  *Composition of teams*: Developing consistent teams of RNs and NAs who are scheduled and partnered consistently together is one strategy to foster teamwork and communication (Campbell et al., 2020).  *Composition of teams*: The team is currently working on dividing themselves into smaller units in an effort to reduce the number of different individuals they work with to create trust, develop the culture necessary to function as a high performing team and to be able to monitor one another's performance, give feedback, conduct closed loop communication, put the team above the individual, and provide the team leadership needed (Kalisch et al., 2007).  *Composition of teams*: Co-workers are a unique source of support, as they are familiar with the medical world and often the specific situation. Additionally, because a majority of the sample had observed or been involved in an error or adverse event, co-workers are probably to have undergone similar experiences themselves. However, healthcare providers also desire other forms of support (i.e. supervisor, institutional). Creating stable teams can ensure the support is there (Winning et al., 2018).  *Composition of teams:* Increased familiarity of team members and the extent to which individuals know the strengths, vulnerabilities, and idiosyncrasies of all the other members, increases productivity and effectiveness. Essentially, people can learn about each other faster if there are fewer people. The ability to offset vulnerabilities or magnify the strengths of one another depends on team members being familiar with one another. As the group size grows, the probability of synergistic behaviour, which requires a thorough knowledge of other team members_ strengths and ways of practicing, declines. Physical proximity allows team members to offset each other’s vulnerabilities and magnify each other’s strengths to a much greater extent than physical distance. Physical distance, on the other hand, reduces the possibilities for coordinated action and division of labour dominates over teamwork. Synergistic cooperation, a basis for horizontal effectiveness, is minimised (Kalisch et al., 2007). |
|  | *Creating time and space for collaboration*: Organising forums for care workers to communicate, by changing the work schedule in cases of staff shortages and by making decisions when conflicts arose in the care worker group. The importance of management was especially verbalised when care workers faced situations where the collaboration between care workers had failed..... Meetings and care plans ensured a common ground for providing care, but should be used as a basis for continued communication rather than a set protocol for care. Only through continued communication, the care workers could create the shared goals and shared knowledge necessary for good collaboration (Jakobsen et al., 2020).  *Creating time and space for collaboration:*  - closed-loop communication implies sending and receiving information and following up to ensure the sent message was actually received as intended. Participants described both formal and informal communication to ensure this happened (Bellury et al., 2016).  *Creating time and space for collaboration:* Another important antecedent of collaboration is an opportunity to collaborate. Nurses depend on leaders to encourage collaboration, optimize work schedules, and structure work environments that facilitate collaboration. Along with the opportunity to collaborate, nurses need a precipitating event that causes collaboration. In nursing, this event is often a patient‐related problem or a health system problem that one cannot solve alone (Schirm et al., 2000).  *Creating time and space for collaboration:* Having policies and procedures in place that assist with determining workload, assignment, delegation, supervision, staffing, skill mix, team composition, and size were viewed as important contributor to creating collaborative practice (Moore et al., 2019).  *Creating time and space for collaboration*: Managers should provide time and opportunity for nurses to meet, build, and maintain collaborative relationships. This could be accomplished by extending regular staff meetings to include informal, social opportunities. Developing a tool that could assess intra-professional collaborative practice should be a priority. Using the tool to address interpersonal skills would be helpful when providing informal feedback and during nurses’ performance reviews (Moore et al., 2019).  *Creating time and space for collaboration:* In order to achieve interdependent collaboration, it was decided a get together every day to ask whether they had cooperated well and then take the opportunity to talk about any problems had arisen in this regard. To achieve open communication and team support, the participants wanted to better use the facility unit meetings. They felt that this meeting should be a forum for expressing their opinions and thoughts and where they could reflect on and share their experiences of troubled conscience. They also felt that the meeting should be a forum, wherein they were allowed to discuss and reach consensus about the team’s objectives regarding the care provided (Ericson-Lidman and Strandberg, 2015).  *Creating time and space for collaboration:* PART of the intervention: Huddles - Problem solving; ad hoc planning to re-establish situation awareness. Reinforcing plans already in place and assessing the need to adjust plans. Debriefs - Process improvement; informal information exchange session designed to improve team performance and effectiveness. An after-action review. Feedback - Information provided for the purpose of improving team performance. It should be timely, respectful, specific, directed towards improvement and considerate (Thomas) |
|  | *Organisation of care*: It is important for nurse managers to consider how the unit is designed and how patient assignments are made to support the collaborative work system of RNs and NAs (Campbell et al., 2020).  *Organisation of care – including everyone in decisions:* Future interventions should be aimed at restructuring RN and NA education, unit redesign and creation of safe work environments. Shift report is another component of unit design that can be modified to enhance communication and teamwork among RNs and NAs. Evidence supports bedside shift report enhances teamwork and communication. However, the inclusion of the NA in shift report has yet to become standard practice (Campbell et al., 2020).  *Organisation of care – including everyone in decisions:* One practice implication is that nursing home care may be improved with explicit assessments and inclusive conversations in nursing homes which seek to strengthen manager and staff relationships, particularly efforts to increase information exchange and shared sense-making about developments in resident care. Nursing assistant empowerment was related to improved nursing assistant performance and coordination with nurses (Toles and Anderson, 2011).  *Organisation of care – including everyone in decisions:* The sub‐theme of shared decision‐making highlighted the importance of mutual goal setting, negotiation, and making joint decisions that positively impacted on collaborative practice (Moore et al., 2019).  *Organisation of care – including everyone in decisions*: The goal of open communication and team support was successfully achieved by changing the content of the facility-unit meetings. With the manager present, they could initiate a discussion about equal care and individual residents’ needs as well as about other important matters such as various perspectives of teamwork and challenging care situations that could generate troubled conscience. They expressed satisfaction with the meeting format and felt that this had helped ease their conscience. However, they still felt abandoned and alone in their struggles with problems in their work environment as well as in their daily caring for residents with severe multi-morbidity and dementia (Ericson-Lidman and Strandberg, 2015).  *Organisation of care – including everyone in decisions*: The more employees participate in decision-making the more likely they are to invest in the outcomes of those decisions and to offer ideas for new and improved ways of working (Heponiemi et al., 2012).  *Organisation of care – including everyone in decisions:* The results of this study show a significant positive relationship between inclusion (e.g., perceiving to be included by one’s supervisor, co-workers and other clinicians) and staff empowerment. Empowered employees who feel heard, feel control over their work and confident in their abilities to perform good care (QOC) while believing that what they do impacts their organization (QOW). (Barry et al., 2019) |
|  | *Provider support:* Ownership type was associated with support for innovation and vision. Our results showed that the ownership type may have an impact on team climate. Team climate levels related to participative safety, vision and support for innovation were higher in not-for-profit organisations (both sheltered homes and nursing homes) compared to for-profit sheltered homes and public sheltered homes. For-profit organizations can be assumed to focus on economic efficiency, minimizing costs and maximizing profit for shareholders, which perhaps is not an ideal environment for the development of good team climate. Instead, not-for-profit sector can be seen guided by an ideological mission and according to our results also has more potential to build a good team climate. Similar kind of results have been found previously, for example, it has been found that in long-term care the satisfaction with supervision levels is lower in employees from for-profit organizations compared to those from not-for-profit organisations. |

| **Supplementary Table 4: Included studies** | | | | | | | |
| --- | --- | --- | --- | --- | --- | --- | --- |
| **No** | **Author** | **Design** | **Country** | **Setting/**  **participants** | **Participants** | **Concept studied** | **Contribution to COM-B model** |
| Intervention studies | | | | | | | |
| 1 | Jakobsen et al (2020) Can a participatory organizational intervention improve social capital and organizational readiness to change? Cluster randomized controlled trial at five Danish hospitals | Randomised controlled trial | Denmark | Hospitals | RNs | Social capital | Social support  Leadership |
| 2 | Thomas et al (2013) Building a culture of safety through team training and engagement | A quasi experimental, interrupted time-series design | USA | Hospitals | RNs | Group cohesion | Culture  Feeling heard  Time/space |
| 3 | Colon-Emeric et al (2014) Connecting the Learners: Improving Uptake of a Nursing Home Educational Program by Focusing on Staff Interactions | Intervention (pre-post test) | USA | Care homes | RNs and care workers | Connections, communication | Being willing  Culture  Feeling heard  Social competence |
| 4 | DeMiglo et al (2005) Group Cohesion and Nurse Satisfaction | Intervention (pre-post test) | USA | Hospitals | RNs | Teamwork, cohesion | Attributes of person  Social competence  Composition of team  Leadership |
| 5 | Kalish et al (2007) An Intervention to Enhance Nursing Staff Teamwork and Engagement | Intervention (pre-post test) | USA | Hospitals | RNs, LPNs, care workers | Teamwork | Attributes of person  Social support  Composition of team  Leadership |
| 6 | Kile et al (2018) Teach nurses to recognize incivility, confront it using cognitive rehearsal techniques, thereby improving job satisfaction. | Intervention (pre-post test) | USA | Hospitals | RNs | Communication | Social competence  Leadership |
| 7 | Ericson-Lidman et al (2015) Learning to deal constructively with troubled conscience related to care providers' perceptions of deficient teamwork in residential care of older people. | Participatory Action Research | Sweden | Care homes | RNs, care workers | Teamwork | Attributes of person  Being willing  Culture  Feeling heard  Leadership |
| 8 | Phan et al (2022) Promoting Civility in the Workplace: Addressing Bullying in New Graduate Nurses Using Simulation and Cognitive Rehearsal. | Intervention (pre-post test) | USA | Hospitals | RNs | Civility, cognitive rehearsal | Social competence  Culture |
| 9 | Lundholm et al (2022) A medical escape room to build intern workplace social capital in an internal medicine residency program | Intervention (pre-post test) | USA | Hospitals | RNs | Social capital | Time space  Social competence |
| Descriptive studies | | | | | | | |
| 10 | Havig et al (2013) Real teams and their effect on the quality of care in nursing homes | Mixed methods (interviews, questionnaires) | Norway | Hospitals | RNs | Teamwork | Social support  Leadership |
| 11 | Hoare et al (2013) Becoming willing to role model. Reciprocity between new graduate nurses and experienced practice nurses in general practice in New Zealand: A constructivist grounded theory | Qualitative (interviews and observation) | NZ | General practice | RNs | Reciprocity | Leadership  Culture  Being willing  Moral support  Social competence  Composition of team |
| 12 | Kim and Oh (2016) Assimilating to Hierarchical Culture: A Grounded Theory Study on Communication among Clinical Nurses | Qualitative (interviews) | South Korea | Hospitals | RNs | Communication | Leadership  Culture  Behaviours  Attributes of care worker  Social competence |
| 13 | Duddle et al (2007) Intra- professional relations in nursing | Qualitative (multiple case study) | Australia | Hospitals | RNs | Relations | Attributes of care worker  Being willing  Moral support  Social competence  Resilience |
| 14 | Padgett et al (2013) Professional collegiality and peer monitoring among nursing staff | Qualitative (interviews and observation) | USA | Hospitals | RN’s | Collegiality | Culture  Being willing  Social competence |
| 15 | Madden et al (2017) Rules of performance in the nursing home: A grounded theory of nurse-CNA communication. | Qualitative (interviews and observation) | USA | Care homes | Care workers | Communication,  Collegiality | Culture,  Respect  Being willing  Feeling heard  Moral support  Social competence  Leadership  Trust |
| 16 | Lux et al (2014) Ending disruptive behaviour: Staff nurse recommendations to nurse educators | Qualitative (interviews) | USA | Hospitals | RNs | Teamwork | Culture  Moral support  Social competence  Leadership  Role modelling |
| 17 | Schirm et al (2000) Caregiving in Nursing Homes Views of Licensed Nurses and Nursing Assistants | Qualitative (interviews) | USA | Hospitals | RNs and LPNs | Teamwork | Values  Being willing  Social support  Social competence  Leadership  Attributes of person |
| 18 | Munkejord et al (2019) Challenging the ethnic pyramid: Golden rules and organisational measures towards a more inclusive work environment | Qualitative (interviews) | Norway | Hospitals | RNs | Collaboration | Leadership  Culture |
| 19 | Potter et al (2004) Understanding RN and Unlicensed Assistive Personnel Working Relationships in Designing Care Delivery Strategies | Qualitative (interviews, focus groups) | USA | Hospitals | RNs | Working relationships | Culture |
| 20 | Jakobsen et al (2018) Collaboration among eldercare workers: barriers, facilitator sand supporting processes. | Qualitative (interviews and focus groups) | Denmark | Care homes | Care workers and RNs | Collaboration | Culture  Attributes of person  Being willing  Feeling heard  Social support  Leadership  Trust  Time/space |
| 21 | Norikoshi et al (2017) A qualitative study on the attributes of nurses’ workplace social capital in Japan | Qualitative (interviews) | Japan | Hospitals | RNs | Social capital | Attributes of person  Being willing  Feeling heard  Social support  Social competence  Composition of teams  Leadership  Trust |
| 22 | Bellury et al (2016) Teamwork in Acute Care: Perceptions of Essential but Unheard Assistive Personnel and the Counterpoint of Perceptions of Registered Nurses | Qualitative (interviews) | USA | Hospitals | RNs, care workers | Teamwork, communication | Attributes of person  Culture  Feeling heard  Social competence  Leadership  Trust  Time and space |
| 23 | Killmeck et al (2021) Changes in Safety and Teamwork Climate After Adding Structured Observations to Patient Safety Walk Rounds. | Qualitative (observational) | Switzerland | Hospitals | RNs | Team work  Communication | Time and space  Culture |
| 24 | Heponiemi et al (2011) Ownership type and team climate in elderly care facilities: the moderating effect of stress factors | Cross-sectional | Finland | Care homes | RNs, care workers | Teamwork | Feeling heard  Culture  Leadership |
| 25 | Barry et al (2019) Team inclusion and empowerment among nursing staff in long-term care. | Cross-sectional | USA | Care homes | RNs, care workers | Teamwork | Culture  Feeling heard  Leadership |
| 26 | Brunetto et al (2013) The importance of supervisor–nurse relationships, teamwork, wellbeing, affective commitment and retention of North American nurses | Cross-sectional | Australia | Hospitals | RNs | Teamwork | Leadership |
| 27 | Winning et al (2017) The emotional impact of errors or adverse events on healthcare providers in the NICU: The protective role of co-worker support | Cross-sectional | USA | Hospitals | RNs | Co-worker support | Attributes of person  Social support  Leadership  Culture  Trust |
| 28 | Lehmann-Willenbrock et al (2012) Appreciating age diversity and German nurse well-being and commitment: Co-worker trust as the mediators. | Cross-sectional | Germany | Hospitals | RNs | Co-worker trust | Attributes of person  Culture  Feeling heard  Social support  Social competence  Leadership  Trust |
| 29 | Quoidbach et al (2009) The impact of trait emotional intelligence on nursing team performance and cohesiveness | Cross-sectional | Belgium | Hospitals | RNs | Cohesion | Attributes of person  Being willing  Social competence  Leadership  Resilience |
| 30 | Yan et al (2022) The social support, psychological resilience and quality of life of nurses in infectious disease departments in China: A mediated model. | Cross-sectional | China | Hospitals | RNs | Social support, resilience | Social competence – |
| 31 | Kaya et al (2022) The relationships between nurses’ positive psychological capital, and their employee voice and organizational silence behaviors | Cross-sectional | Turkey | Hospitals | RNs | Psychological capital | Social competence – |
| 32 | Pedersen et al (2023) Positive association between social capital and the quality of health care service: A cross-sectional study. | Cross-sectional | Denmark | Hospitals | Hospital employee | Social capital, bonding | Social competence |
| 33 | Velando-Soriano et al (2018) Impact of social support in preventing burnout syndrome in nurses: A systematic review | Literature review | Spain | Hospitals | RNs | Social support | Culture  Feeling heard  Social support  Leadership |
| 34 | Campbell et al (2020) Interventions to promote teamwork, delegation and communication among registered nurses and nursing assistants: An integrative review | Literature review | USA | Hospitals | RNs and care workers | Teamwork communication | Attributes of person  Being willing  Culture  Leadership  Trust |
| 35 | Toles et al (2011) State of the science: Relationship-oriented management practices in nursing homes | Literature review | USA | Care homes | RNs, care workers | Relationships | Culture  Leadership |
| 36 | Moore et al (2019) Collaboration among registered nurses and practical nurses in acute care hospital: a scoping review | Literature review | USA | Hospitals | RNs | Collaboration | Being willing  Culture  Feeling heard  Social competence  Leadership  Trust  Time/space |
| 37 | Kalisch et al (2010) The Development and Testing of the Nursing Teamwork Survey | Measurement | USA | Hospitals | RNs | Teamwork | Composition of team  Leadership |
| 38 | Kalisch et al (2005) Improving Nursing Unit Teamwork | Opinion | USA | Hospitals | RNs | Teamwork | Being willing  Social support  Social competence  Leadership |
| 39 | Hofmeyer et al (2008) Building social capital in healthcare organizations: Thinking ecologically for safer care | Opinion | Canada | Hospitals | RNs | Social capital | Attributes of person  Culture  Social support  Leadership  Time/space  Composition of teams |
| 40 | Roth et al (2011) Essentials for Great Teams: Trust, Diversity, Communication ... and Joy | Opinion | USA | Hospitals | RNs | Teamwork | Attributes of person  Being willing  Culture  Feeling heard  Social competence  Leadership  Trust |
| 41 | Clark et al (2022) Civility: A concept analysis revisited | Concept analysis | USA | Hospitals | RNs | Civility  Respect | Social competence  Leadership  Time/space  Being willing |
| 42 | Emich et al (2018) Conceptualizing collaboration in nursing | Concept analysis | USA | Hospitals | RNs | Collaboration  Respect  Teamwork  Sharing | Social competence  Leadership  Culture  Being willing |

References

BARRY, T. T., LONGACRE, M., CARNEY, K. O. S. & PATTERSON, S. 2019. Team inclusion and empowerment among nursing staff in long-term care. *Geriatric nursing (New York),* 40**,** 487-493.

BELLURY, L., HODGES, H., CAMP, A. & ADUDDELL, K. 2016. Teamwork in Acute Care: Perceptions of Essential but Unheard Assistive Personnel and the Counterpoint of Perceptions of Registered Nurses: NAP-RN PERCEPTIONS OF TEAMWORK. *Research in nursing & health,* 39**,** 337-346.

BRUNETTO, Y., SHRIBERG, A., FARR-WHARTON, R., SHACKLOCK, K., NEWMAN, S. & DIENGER, J. 2013. The importance of supervisor-nurse relationships, teamwork, wellbeing, affective commitment and retention of North American nurses. *Journal of nursing management,* 21**,** 827-837.

CAMPBELL, A. R., LAYNE, D., SCOTT, E. & WEI, H. 2020. Interventions to promote teamwork, delegation and communication among registered nurses and nursing assistants: An integrative review. *Journal of nursing management,* 28**,** 1465-1472.

COLÓN-EMERIC, C. S., PINHEIRO, S. O., ANDERSON, R. A., PORTER, K., MCCONNELL, E., CORAZZINI, K., HANCOCK, K., LIPSCOMB, J., BEALES, J. & SIMPSON, K. M. 2014. Connecting the Learners: Improving Uptake of a Nursing Home Educational Program by Focusing on Staff Interactions. *The Gerontologist,* 54**,** 446-459.

DIMEGLIO, K., PADULA, C., PIATEK, C., KORBER, S., BARRETT, A., DUCHARME, M., LUCAS, S., PIERMONT, N., JOYAL, E., DENICOLA, V. & CORRY, K. 2005. Group Cohesion and Nurse Satisfaction: Examination of a Team-Building Approach. *The Journal of nursing administration,* 35**,** 110-120.

DUDDLE, M. & BOUGHTON, M. 2007. Intraprofessional relations in nursing. *Journal of advanced nursing,* 59**,** 29-37.

ERICSON-LIDMAN, E. & STRANDBERG, G. 2015. Learning to deal constructively with troubled conscience related to care providers' perceptions of deficient teamwork in residential care of older people - a participatory action research study. *Scandinavian journal of caring sciences,* 29**,** 215-224.

HEPONIEMI, T., ELOVAINIO, M., KOUVONEN, A., NORO, A., FINNE-SOVERI, H. & SINERVO, T. 2012. Ownership type and team climate in elderly care facilities: the moderating effect of stress factors: Ownership type and team climate in elderly care. *Journal of advanced nursing,* 68**,** 647-657.

HOARE, K. J., MILLS, J. & FRANCIS, K. 2013. Becoming willing to role model. Reciprocity between new graduate nurses and experienced practice nurses in general practice in New Zealand: A constructivist grounded theory. *Collegian (Royal College of Nursing, Australia),* 20**,** 87-93.

HOFMEYER, A. & MARCK, P. B. 2008. Building social capital in healthcare organizations: Thinking ecologically for safer care. *Nursing outlook,* 56**,** 145.e1-145.e9.

JAKOBSEN, L. M., ALBERTSEN, K., JORGENSEN, A. F. B., GREINER, B. A. & RUGULIES, R. 2018. Collaboration among eldercare workers: barriers, facilitators and supporting processes. *Scandinavian journal of caring sciences,* 32**,** 1127-1137.

JAKOBSEN, M. D., CLAUSEN, T. & ANDERSEN, L. L. 2020. Can a participatory organizational intervention improve social capital and organizational readiness to change? Cluster randomized controlled trial at five Danish hospitals. *Journal of advanced nursing,* 76**,** 2685-2695.

KALISCH, B. J. & BEGENY, S. M. 2005. Improving Nursing Unit Teamwork. *The Journal of nursing administration,* 35**,** 550-556.

KALISCH, B. J., CURLEY, M. & STEFANOV, S. 2007. An Intervention to Enhance Nursing Staff Teamwork and Engagement. *The Journal of nursing administration,* 37**,** 77-84.

KILE, D., EATON, M., DEVALPINE, M. & GILBERT, R. 2019. The effectiveness of education and cognitive rehearsal in managing nurse‐to‐nurse incivility: A pilot study. *Journal of nursing management,* 27**,** 543-552.

KIM, M. & OH, S. 2016. Assimilating to Hierarchical Culture: A Grounded Theory Study on Communication among Clinical Nurses. *PloS one,* 11**,** e0156305-e0156305.

LEHMANN-WILLENBROCK, N., LEI, Z. & KAUFFELD, S. 2012. Appreciating age diversity and German nurse well-being and commitment: Co-worker trust as the mediator. *Nursing & health sciences,* 14**,** 213-220.

LUX, K. M., HUTCHESON, J. B. & PEDEN, A. R. 2014. Ending disruptive behavior: Staff nurse recommendations to nurse educators. *Nurse education in practice,* 14**,** 37-42.

MADDEN, C., CLAYTON, M., CANARY, H. E., TOWSLEY, G., CLOYES, K. & LUND, D. 2017. Rules of performance in the nursing home: A grounded theory of nurse–CNA communication. *Geriatric nursing (New York),* 38**,** 378-384.

MOORE, J., PRENTICE, D., CRAWFORD, J., LANKSHEAR, S., LIMOGES, J. & RHODES, K. 2019. Collaboration among registered nurses and practical nurses in acute care hospitals: A scoping review. *Nursing forum (Hillsdale),* 54**,** 376-385.

MUNKEJORD, M. C. 2019. Challenging the ethnic pyramid: Golden rules and organisational measures towards a more inclusive work environment. *Journal of nursing management,* 27**,** 1522-1529.

NORIKOSHI, K., KOBAYASHI, T. & TABUCHI, K. 2018. A qualitative study on the attributes of nurses' workplace social capital in Japan. *Journal of nursing management,* 26**,** 74-81.

PADGETT, S. M. 2013. Professional collegiality and peer monitoring among nursing staff: An ethnographic study. *International journal of nursing studies,* 50**,** 1407-1415.

POTTER, P. & GRANT, E. 2004. Understanding RN and Unlicensed Assistive Personnel Working Relationships in Designing Care Delivery Strategies. *The Journal of nursing administration,* 34**,** 19-25.

QUOIDBACH, J. & HANSENNE, M. 2009. The Impact of Trait Emotional Intelligence on Nursing Team Performance and Cohesiveness. *Journal of professional nursing,* 25**,** 23-29.

SCHIRM, V., ALBANESE, T., GARLAND, T. N., GIPSON, G. & BLACKMON, D. J. 2000. Caregiving in Nursing Homes: Views of Licensed Nurses and Nursing Assistants. *Clinical nursing research,* 9**,** 280-297.

THOMAS, L. & GALLA, C. 2013. Building a culture of safety through team training and engagement. *BMJ quality & safety,* 22**,** 425-434.

TOLES, M. & ANDERSON, R. A. 2011. State of the science: Relationship-oriented management practices in nursing homes. *Nursing Outlook,* 59**,** 221-227.

VELANDO‐SORIANO, A., ORTEGA‐CAMPOS, E., GÓMEZ‐URQUIZA, J. L., RAMÍREZ‐BAENA, L., DE LA FUENTE, E. I. & CAÑADAS‐DE LA FUENTE, G. A. 2020. Impact of social support in preventing burnout syndrome in nurses: A systematic review. *Japan journal of nursing science : JJNS,* 17**,** e12269-n/a.

WINNING, A. M., MERANDI, J. M., LEWE, D., STEPNEY, L. M. C., LIAO, N. N., FORTNEY, C. A. & GERHARDT, C. A. 2018. The emotional impact of errors or adverse events on healthcare providers in the NICU: The protective role of coworker support. *Journal of advanced nursing,* 74**,** 172-180.
